# Ent3 and GGA adaptors facilitate diverse anterograde and retrograde trafficking events to and from the prevacuolar endosome

**DOI:** 10.1038/s41598-019-47035-5

**Published:** 2019-07-24

**Authors:** Francisco Yanguas, Esteban Moscoso-Romero, M.-Henar Valdivieso

**Affiliations:** 1 Departamento de Microbiología y Genética, Universidad de Salamanca, Salamanca, Spain; 2 Instituto de Biología Funcional y Genómica (IBFG), Consejo Superior de Investigaciones Científicas (CSIC), Calle Zacarías González 2, 37007 Salamanca, Spain

**Keywords:** Fungal biology, Endosomes

## Abstract

Carboxypeptidases Y (Cpy1) and S (Cps1), the receptor Vps10, and the ATPase subunit Vph1 follow the carboxypeptidase Y (CPY) pathway from the trans-Golgi network (TGN) to the prevacuolar endosome (PVE). Using *Schizosaccharomyces pombe* quantitative live-cell imaging, biochemical and genetic analyses, we extended the previous knowledge and showed that collaboration between Gga22, the dominant Golgi-localized Gamma-ear-containing ARF-binding (GGA) protein, and Gga21, and between Gga22 and the endosomal epsin Ent3, was required for efficient: i) Vps10 anterograde trafficking from the TGN to the PVE; ii) Vps10 retrograde trafficking from the PVE to the TGN; iii) Cps1 exit from the TGN, and its sorting in the PVE *en* route to the vacuole; and iv) Syb1/Snc1 recycling to the plasma membrane through the PVE. Therefore, monomeric clathrin adaptors facilitated the trafficking of Vps10 in both directions of the CPY pathway, and facilitated trafficking events of Cps1 in different organelles. By contrast, they were dispensable for Vph1 trafficking. Thus, these adaptors regulated the traffic of some, but not all, of the cargo of the CPY pathway, and regulated the traffic of cargoes that do not follow this pathway. Additionally, this collaboration was required for PVE organization and efficient growth under stress.

## Introduction

Alterations in the homeostasis of the endosomal-lysosomal system are related to human disease, and protein delivery to the lysosome (vacuole in yeast) is essential for maintaining homeostasis^1^. Several membranous compartments participate in this delivery. According to Day *et al*.^2^, the yeast endosomal system is limited to the trans-Golgi network (TGN), which includes the organelles previously termed late Golgi and early endosomes, and the prevacuolar endosome (PVE), which refers to the late endosomes and multivesicular bodies. Membrane proteins endocytosed at the cell surface and destined for vacuolar degradation reach the TGN and later the PVE, where they are internalized into intraluminal vesicles in a process mediated by the endosomal sorting complexes required for transport (ESCRT). Finally, they are released into the vacuolar lumen where they are exposed to proteases^3^.

Vacuolar hydrolases, such as the carboxypeptidase Y (Prc1/Cpy1), proteinase A, and carboxypeptidase S (Cps1) are synthesized in the endoplasmic reticulum, before being transported to the TGN. There, they are diverted from the general secretory pathway to the endocytic pathway by transmembrane receptors and soluble adaptors (reviewed in^3–7^). Vps10 is the receptor that interacts with Cpy1 and proteinase A, which are soluble proteins, ensuring their sorting into clathrin-coated vesicles destined for the PVE. From the PVE, Cpy1 is released into the vacuole where it undergoes proteolytic processing, while Vps10 is recycled back to the TGN by the retromer^8,9^. Mutations that interfere with Vps10 trafficking result in defects in Cpy1 processing and cause its missorting to the cell surface. Cps1 is a transmembrane protein that reaches the PVE in a Vps10-independent manner^4,8,10,11^. The trafficking route from the TGN to the PVE is known as the CPY pathway.

The sorting of transmembrane receptors that follow the CPY pathway requires the participation of Golgi-localized Gamma-ear-containing ARF-binding (GGA) clathrin adaptors^12–16^. Although adaptor protein-1 (AP-1) and GGAs participate in cargo sorting in the TGN, the relationship between the two types of adaptors is complex^16–22^. Epsins are clathrin-associated monomeric adaptors with a conserved role in endocytosis^23^. Ent3 and Ent5 are *Saccharomyces cerevisiae* endosomal proteins that have an epsin N-terminal homology (ENTH) domain, interact with clathrin, and participate in protein sorting. In particular, Ent3 is functionally related to the GGAs, while Ent5 interacts with Gga2 but is more related to AP-1 function^24–28^.

Substantial information about the CPY pathway was obtained indirectly, analyzing *S. cerevisiae* Cpy1 processing and missorting, or using chimeras as model cargoes. In this study, we used live-cell imaging of *Schizosaccharomyces pombe* Vps10-GFP, Cpy1-Cherry, Ub:GFP-Cps1 and Vph1-GFP, expressed from the chromosome, to revisit the participation of different adaptors in this pathway. Our results were consistent with many of the previous hypotheses regarding the involvement of AP-1, GGAs and endosomal epsins in the process. They also provided additional information on the collaboration between the GGAs, and between Gga22 (the dominant GGA) and the endosomal epsin Ent3 in multiple anterograde and retrograde trafficking events involving the PVE. Thus, our results argued against the principle One cargo-One adaptor-One route. Finally, we showed that the integrity of the PVE and efficient cell growth under stress conditions depend on collaboration between monomeric clathrin adaptors.

## Results

### Analysis of the involvement of AP-1 and GGAs in Vps10 trafficking by live-cell imaging

**Figure 1:**
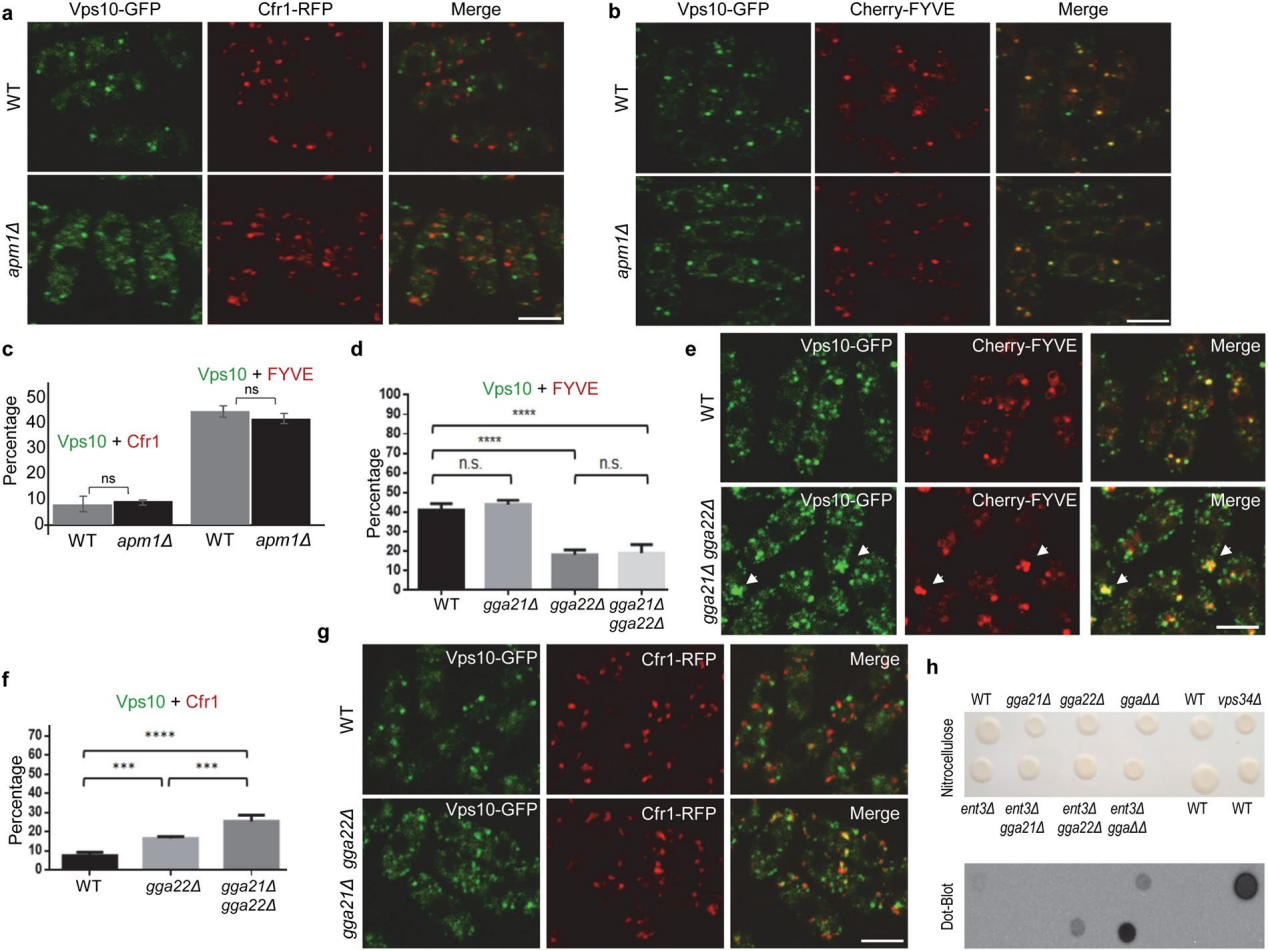
Analysis of Vps10-GFP trafficking in adaptor mutants. **(a)** Wild-type (WT) and *apm1Δ* cells containing Vps10-GFP and Cfr1-RFP. **(b)** WT and *apm1Δ* cells containing Vps10-GFP and Cherry-FYVE. **(c)** Quantification of the colocalization between Vps10-GFP and Cfr1-RFP or Cherry-FYVE. A minimum of 500 GFP dots from each of three independent experiments were scored. For each value, the mean, standard deviation, and statistical significance determined using the t-test are shown. ns, non-significant. **(d)** Percentage of Vps10-GFP colocalization with Cherry-FYVE in WT, *gga21Δ*, *gga22Δ*, and *gga21Δ gga22Δ* cells. **(e)** WT and *gga21Δ gga22Δ* cells containing Vps10-GFP and Cherry-FYVE. The arrowheads denote enlarged endosomes. **(f)** Percentage of Vps10-GFP colocalization with Cfr1-RFP in WT, *gga22Δ*, and *gga21Δ gga22Δ* cells. **(g)** WT and *gga21Δ gga22Δ* cells containing Vps10-GFP and Cfr1-RFP. **(h)** Upper panel, colonies from the indicated strains were grown on a nitrocellulose filter deposited onto a minimal medium plate and photographed (WT, wild-type. *ggaΔΔ*, *gga21Δ gga22Δ*). The cells were washed and the filter was subjected to anti-Cpy1 immunoblotting (lower panel). In (**a**,**b**,**e**,**g**) images are single planes, captured with a confocal spinning-disk microscope. Bar, 10 µm. In (**d**,**f**), a minimum of 350 GFP dots from each of a minimum of three experiments were scored. The mean, standard deviation, and statistical significance, determined by the Sidak’s (**d**) and Tukey’s (**f**) multiple comparisons tests after analysis of variance (ANOVA), are shown for each value. ***p < 0.001; ****p < 0.0001.

Vps10 is a model cargo to study transport routes between the TGN and the PVE. In order to get direct information about the involvement of clathrin adaptors in its trafficking, we analyzed Vps10-GFP localization by live-cell microscopy using a confocal spinning-disk microscope. In *S. pombe*, Vps10-GFP was observed as discrete fluorescent dots in the cytoplasm^8,29^. We performed colocalization studies using the exomer component Cfr1-RFP, which acted as a stable TGN marker (Fig. S1), and the phosphatidylinositol 3-phosphate (PI3P) probe Cherry-FYVE as a PVE marker^2,30^. This showed that a minor fraction of the Vps10-GFP dots (7.12 ± 2.07%) localized at the TGN, while most of them (41.60 ± 2.66%) localized at the PVE (Fig. 1). Additionally, several tiny fluorescent dots that did not colocalize with Cfr1 or FYVE were observed; therefore, their nature was unknown. They might correspond to fragmented maturing endosomes, with few Cfr1 molecules and whose low level of PI3P would not allow efficient Cherry-FYVE binding. This would explain why less than 50% of the Vps10 dots colocalized with a marker. The preferential localization at the PVE was unexpected, because in budding yeast most Vps10 localizes at the TGN^11,31–33^. The fact that the *S. pombe* receptor was observed at the vacuole membrane in retromer mutants^8^, showed that in fission yeast Vps10-GFP cycles between the TGN and the PVE, as also described in budding yeast^9^. The reason why Vps10 steady-state localization was different in both organisms was unknown, although it might be related to the fact that the carboxy-terminal tail is the least conserved region of both orthologues^8^. This is the part of the protein that bears the potential Golgi retention signals^8,31,34^ (FYVF and YSSL in budding yeast, and FSSIPIFF in fission yeast). It was possible that the *S. pombe* signal was less efficient for retromer recognition than that of *S. cerevisiae*. In any case, this dual localization facilitated a detailed analysis of the factors that influenced Vps10 anterograde transport, by quantifying its colocalization with the TGN and PVE markers in different mutants.

Following this experimental strategy, we determined the involvement of AP-1 in Vps10 transport. The level of colocalization between Vps10-GFP and Cfr1-RFP, and between Vps10-GFP and Cherry-FYVE was similar in WT and *apm1Δ* cells (Fig. 1a–c). This indicated that AP-1 did not play a substantial role in Vps10 exit from the TGN, as described previously using other experimental approaches in budding yeast^21,35^.

Next, we analyzed the contribution of GGA adaptors to Vps10 exit from the TGN. *S. pombe* genome sequencing and curation identified two sequences (*gga21*^+^ and *gga22*^+^) similar to *S. cerevisiae GGA2* (Pombase^36,37^. https://www.pombase.org/). The *gga21Δ* deletion did not alter Vps10 localization at the PVE, as determined by its colocalization with Cherry-FYVE (Figs 1d and S1b). On the contrary, there was a significant reduction in Vps10/FYVE colocalization in the *gga22Δ* mutant, and when both GGA genes were deleted (18.22 ± 2.39% and 19.06 ± 4.34% of the Vps10-GFP dots colocalized with Cherry-FYVE in the *gga22Δ* and *gga21Δ gga22Δ* strains, respectively. Thus, in the mutants, coincidence of both markers was reduced to approximately 45% of the value for the control. Figures 1d,e and S1b). To complement these results, we analyzed the colocalization between Vps10-GFP and Cfr1-RFP (Figs 1f,g and S1c,d). In *gga22Δ* and *gga21Δ gga22Δ* cells, there was an increase in Vps10/Cfr1 coincidence with respect to the control strain (from 7.12 ± 2.07% to 16.49 ± 0.94 and to 25.37 ± 3.28% in the single and double mutants, respectively). In agreement with Vps10 trafficking defects, colony dot-blot showed that in the *gga21Δ gga22Δ* strain the carboxypeptidase was at least partially missorted to the cell surface^30^ (Fig. 1h). Altogether, these results showed that both GGAs were involved in Vps10 exit from the TGN towards the PVE, with one of the adaptors (Gga22) playing a major role, and the other (Gga21), a minor and partially redundant role. Similar conclusions were reached by other groups using different techniques in budding yeast^13–15,21^, which validate our experimental approach.

### A fraction of Vps10 is sorted to the PVE in the absence of GGAs

**Figure 2:**
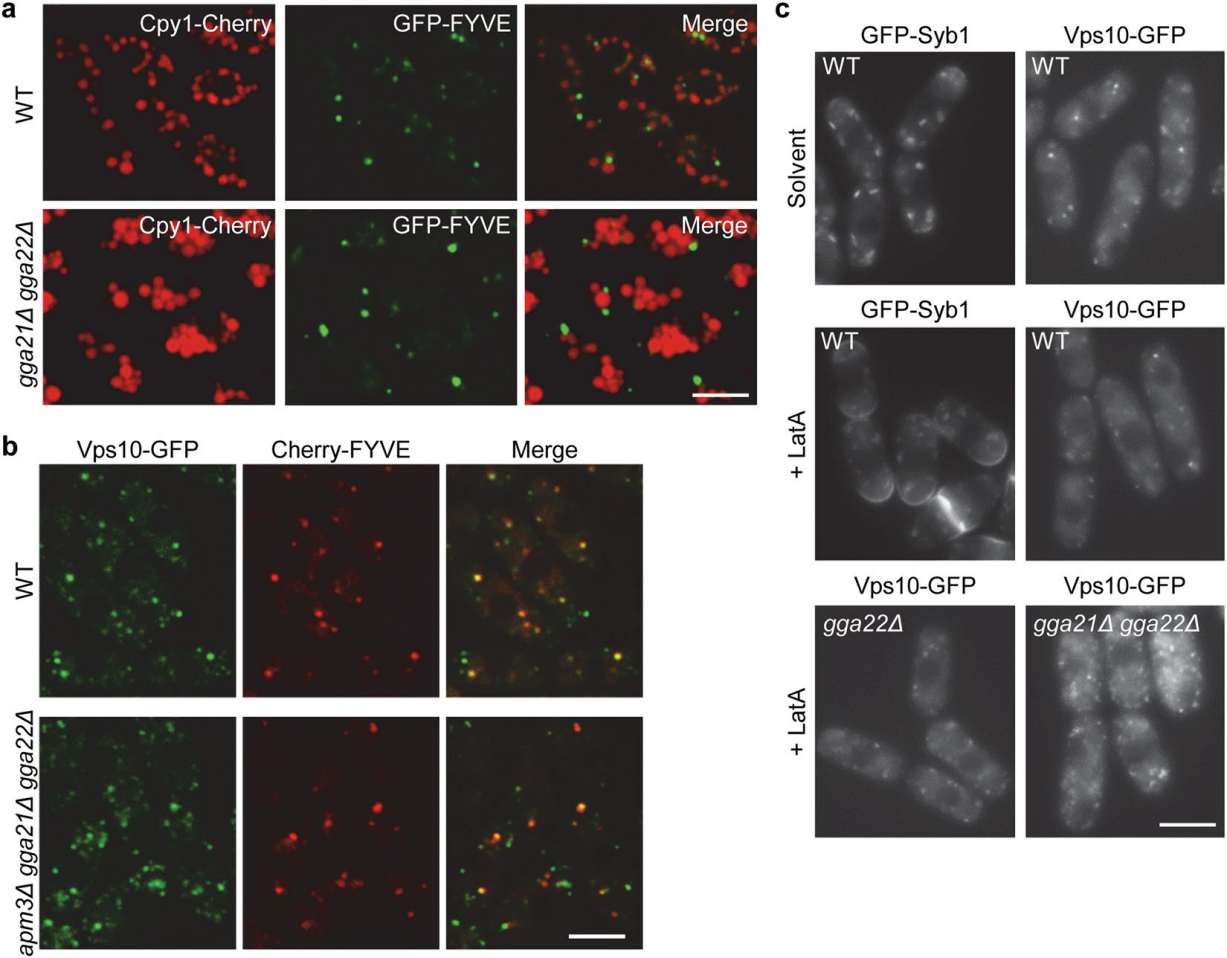
Analysis of the contribution of AP-3 and endocytosis to Vps10 traffic in the absence of GGA proteins. **(a)** Cpy1-Cherry distribution in WT and *gga21Δ gga22Δ* cells containing GFP-FYVE. **(b)** Vps10-GFP colocalization with Cherry-FYVE in WT and *apm3Δ gga21Δ gga22Δ* cells. **(c)** Cells from the indicated strains containing either GFP-Syb1 or Vps10-GFP were treated with DMSO (solvent) and with latrunculin A (+ LatA) for 10 minutes, and photographed. The images in (**a**), and (**b**) are single planes, captured with a confocal spinning-disk microscope, while the images in (**c**) were captured with a Leica conventional fluorescence microscope. Bar, 10 µm.

Colocalization experiments indicated that for the *gga21Δ gga22Δ* mutant Vps10 delivery to the PVE was reduced, but not blocked. To confirm that part of Vps10 reached the PVE, we analyzed Cpy1 distribution, and found that the *gga21Δ gga22Δ* strain exhibited Cpy1-Cherry fluorescence into the vacuole (Fig. 2a), showing that a fraction of the carboxypeptidase reached its final destination in the absence of both GGAs. The hypothesis that in *ggaΔ* mutants part of Vps10 would be retained at the TGN and part would reach the PVE, from where it would recycle, was proposed to explain two apparently contradictory results. In *ggaΔ* mutants Vps10 was observed by immunofluorescence at the TGN, while Cpy1 was secreted^14,16,38^. Our results confirmed this hypothesis.

We performed several experiments to understand the trafficking of Vps10 in the absence of GGA adaptors. After several attempts, one *apm1Δ gga21Δ gga22Δ* clone was obtained; however, it was very difficult to work with it, such that analysis of Vps10 delivery in the absence of AP-1 and the GGAs could not be addressed. We explored the possibility that AP-3, which facilitate transport of alkaline phosphatase (ALP) along the ALP pathway bypassing the PVE^4,39^, mediated Vps10 delivery to the vacuole in the *gga21Δ gga22Δ* strain; based on its colocalization with Cherry-FYVE, Vps10-GFP reached the PVE in the *apm3Δ gga21Δ gga22Δ* strain (Fig. 2b). Finally, it was possible that in the absence of the GGAs, Vps10 was missorted to the plasma membrane, from where it would be endocytosed and delivered to the PVE, as described in clathrin mutants^40^. To address this possibility, endocytosis was inhibited by treating cells with latrunculin A. If the hypothesis was correct, Vps10 should be found at the cell surface in the presence of the drug. As a control, the distribution of GFP-Syb1 was analyzed; Syb1 is the *S. pombe* Snc1 homologue. As shown in Fig. 2c, in the presence of latrunculin A, GFP-Syb1 was observed at the cell surface of cell poles and septal area of the wild-type cells, but Vps10-GFP was not. Similarly, Vps10 was undetectable at the cell surface of *gga22Δ* and *gga21Δ gga22Δ* treated cells. These results demonstrated that Vps10 was not diverted at a significant level to the ALP pathway or the plasma membrane in the absence of both GGA adaptors.

### Ent3 collaborates with Gga22 in anterograde transport from the TGN to the PVE

**Figure 3:**
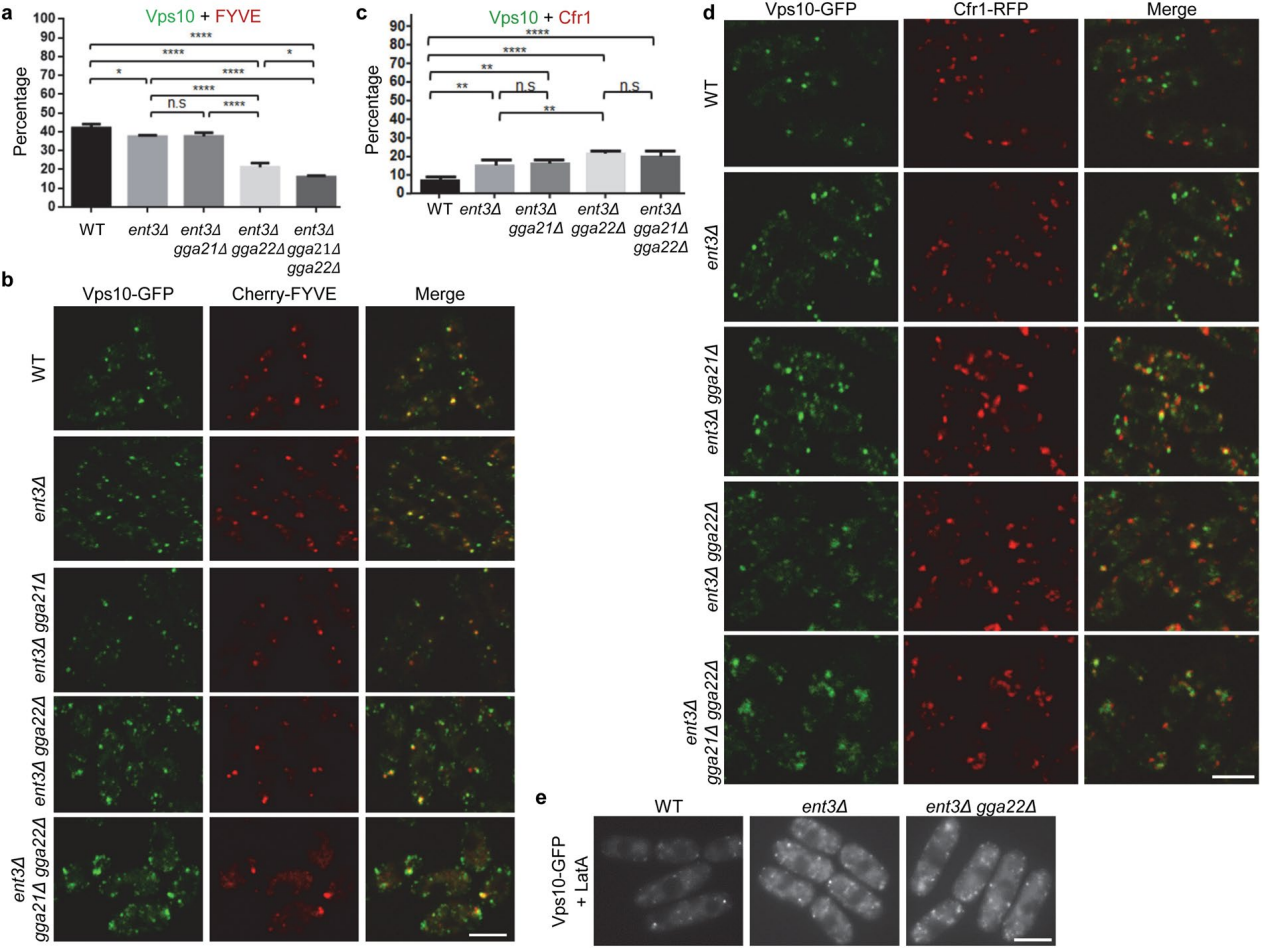
Ent3 participates in the exit of Vps10 from the TGN, and collaborates with Gga22 in this process. **(a)** Percentage of Vps10-GFP colocalization with Cherry-FYVE in wild-type (WT), *ent3Δ*, *ent3Δ gga21Δ*, *ent3Δ gga22Δ*, and *ent3Δ gga21Δ gga22Δ* cells. **(b)** Some of the cells scored in (**a**). **(c)** Percentage of Vps10-GFP colocalization with Cfr1-RFP in WT, *ent3Δ*, *ent3Δ gga21Δ*, *ent3Δ gga22Δ*, and *ent3Δ gga21Δ gga22Δ* cells. **(d)** Some of the cells scored in (**c**). **(e)** Cells from the indicated strains were treated with latrunculin A for 10 minutes. In (**a**,**c**), a minimum of 350 GFP dots from each of a minimum of three experiments were scored. The mean, standard deviation, and statistical significance, determined by the Sidak’s multiple comparisons tests after ANOVA, are shown for each value. *p < 0.05; **p < 0.01; ****p < 0.0001; ns, non-significant. The images in (**b**,**d**) are single planes captured with a confocal spinning-disk microscope, and the images in (**e**) were captured with a Leica conventional fluorescence microscope. Bar, 10 µm.

We analyzed whether Ent3 (the only *S. pombe* endosomal epsin) played a role in Vps10 delivery to the PVE, and whether it collaborated with the GGA adaptors in this process. As for Gga22, most of Ent3 localized at the TGN, according to its colocalization with the phosphatidylinositol-4 phosphate probe Cherry-PH (Fig. S2). In the *ent3Δ* mutant, Vps10/FYVE colocalization was slightly reduced with respect to the values for the control strain (from 42.07 ± 2.08% to 37.5 ± 0.75%. Figure 3a,b) while Vps10/Cfr1 colocalization was increased (from 6.78 ± 2.37 to 14.68 ± 3.16%. Figure 3c,d), indicating a defect in Vps10 exiting the TGN. This defect was consistent with the reduced Cpy1 processing observed in *S. cerevisiae ent3-1* mutant by pulse-chase analysis^28^. The alteration in *S. pombe* Vps10 trafficking was not a spurious consequence of a generalized altered localization of proteins involved in TGN to PVE transport because the localization of Clc1, Apm1, Gga22 and Pep12 was similar in wild-type and *ent3Δ* cells (Fig. S3). In the *ent3Δ gga21Δ* double mutant, Vps10/FYVE colocalization was similar to that in the *ent3Δ* strain (Fig. 3a,b). In the *ent3Δ gga22Δ* and *ent3Δ gga21Δ gga22Δ* mutants, Vps10/FYVE coincidence was reduced from 42.07 ± 2.08% to 21.25 ± 2.18% and to 16.03 ± 0.73%, respectively. Regarding Vps10/Cfr1 colocalization, it was similar in the *ent3Δ gga21Δ* and the *ent3Δ* mutants (Fig. 3c,d). By contrast, Vps10/Cfr1 coincidence increased from 6.78 ± 2.37 up to 21.47 ± 1.36% and to 19.75 ± 3.69% in *ent3Δ gga22Δ* and *ent3Δ gga21Δ gga22Δ* strains, respectively. Exposure to latrunculin A did not result in Vps10 accumulation at the plasma membrane in *ent3Δ* and *ent3Δ gga22Δ* mutants (Fig. 3e). Finally, dot-blot analyses showed that Cpy1 was partially missorted in *ent3Δ gga22Δ*, and that missorting was more severe in *ent3Δ gga21Δ gga22Δ* (Fig. 1h). These results showed that Ent3 participated in Vps10 exiting the TGN and collaborated with Gga22 in this process. Altogether, the results described in this and the previous sections showed that efficient Vps10 transport from the TGN to the PVE was not facilitated by a single clathrin adaptor, but required the presence of Gga22 and either Gga21 or Ent3. Nevertheless, part of the Vps10 was delivered to the PVE in the *ent3Δ gga21Δ gga22Δ* mutant, showing that the adaptors facilitated Vps10 trafficking from the TGN to the PVE but were not essential for this trafficking, where additional mechanisms might participate.

### Cps1 exiting the TGN and sorting to the vacuole lumen is defective in *gga21Δ gga22Δ*, *ent3Δ gga22Δ* and *ent3Δ gga21Δ gga22Δ* mutants

**Figure 4:**
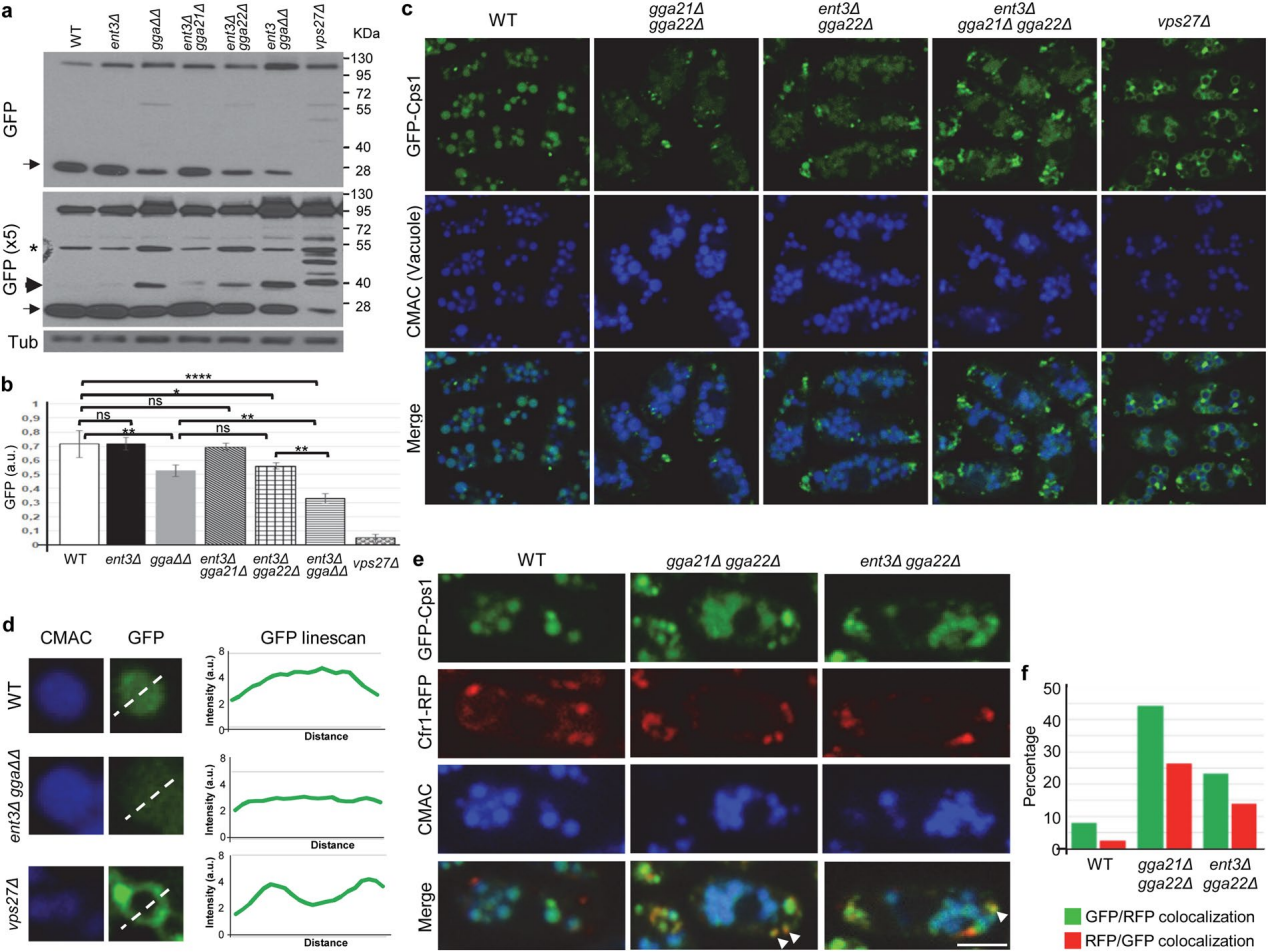
Ggga22 collaborates with Gga21 and with Ent3 in Cps1 trafficking. (**a**) Equal amounts of protein in cell extracts from the indicated strains containing Ub:GFP-Cps1 were subjected to SDS-PAGE, and were immunoblotted with anti-GFP (upper and middle panels) and anti-tubulin (Tub, lower panel; loading control) antibodies. The same blot was exposed for a short (upper panel) or a long (x5; middle panel) time. The asterisk denotes a non-specific band, the arrows denote the GFP band, and the arrowhead denotes a 40-kDa band present in some mutants. WT: wild-type. *ggaΔΔ*: *gga21Δ gga22Δ. ent3Δ ggaΔΔ*: *ent3Δ gga21Δ gga22Δ*. **(b)** The intensity of the GFP band on films from three independent western blots from the same strains as in (a) was quantified with ImageJ software. The mean intensity (a.u.), standard deviation, and statistical significance, determined by Sidak’s multiple comparisons test after ANOVA, are shown for each strain. *p < 0.05; **p < 0.01; ****p < 0.0001; ns, non-significant. **(c)** Distribution of Ub:GFP-Cps1 in different strains. The distribution of Ub:GFP-Cps1 (upper panels), Blue CMAC-stained vacuoles (middle planes) and the merged images (lower panel) is shown. (**d**) Enlargement of representative vacuoles from the indicated strains. The signal corresponding to Blue CMAC (left panels) and to Ub:GFP-Cps1 (central panels) is shown. The right panels show linescans of the GFP fluorescence intensity (a.u.) across the vacuole, as indicated by the dotted line in the central panels. The images are single planes captured with a DeltaVision system. Bar, 10 µm. **(e)** Ub:GFP-Cps1 colocalization with Cfr1-RFP. Vacuolar staining with Blue CMAC is shown. Arrowheads denote GFP-Cps1/Cfr1-RFP colocalization. Images are single planes captured with a confocal spinning-disk microscope. Bar, 5 µm. **(f)** Quantification of Cps1/Cfr1 coincidence. For better accuracy both, the percentage of GFP-Cps1 dots that colocalized with Cfr1-RFP dots and the percentage of Cfr1-RFP dots that colocalized with GFP-Cps1 dots were determined. A minimum of 150 Cps1 dots from a single experiment were scored for each strain.

We analyzed whether the Gga22/Gga21 and Gga22/Ent3 collaboration was specific for Vps10 exiting the TGN, or facilitated the transport of other proteins that populated the CPY pathway. Cps1 is synthesized as an ubiquitinated precursor that is delivered from the TGN to the PVE, from where it reaches the vacuole membrane. There, it is cleaved by hydrolases, resulting in its maturation and release into the vacuole lumen as a soluble protein^4,41^. If Cps1 processing is blocked, the protein remains associated to the vacuole membrane, a situation observed if ubiquitination is reduced and in ESCRT-defective mutants^42–44^. When GFP-Cps1 is released into the vacuole, GFP is clipped by proteolytic cleavage. Therefore, the intensity of a 28 kDa-band detected by western blotting can be used as a proxy for the extent of Cps1 sorting into the vacuole lumen. We used a Ub:GFP-Cps1 fusion protein integrated into the chromosome to analyze *S. pombe* Cps1 processing in different mutants by western blotting. We observed a different pattern of bands in extracts from different strains (Fig. 4a,b). In wild-type, *ent3Δ*, and *ent3Δ gga21Δ* extracts, a strong 28 kD-band was observed. By comparison, the intensity of this band was significantly reduced in *gga21Δ gga22Δ* and *ent3Δ gga22Δ* extracts, showing that in these strains Cps1 sorting to the vacuole was inefficient. The reduction was even greater in *ent3Δ gga21Δ gga22Δ* extracts. Additionally, in the adaptor mutants a subtle band of approximately 40 kDa was observed. This band was stronger in the strains where the GFP band was weaker, and might indicate abnormal Cps1 processing. The *vps27Δ* extract exhibited a pattern of bands that was distinct from those described above. The 28 kDa-band was almost undetectable, in agreement with strong blocking of the release of Cps1 into the vacuole. Additionally several weak bands larger than 40 kDa were also observed in this ESCRT mutant.

Defects in Cps1 sorting at the PVE and in its proteolytic processing have been described in *S. cerevisiae gga1Δ gga2Δ*, *ent3-1* and *ent3Δ ent5Δ* mutants^16,26–28^, showing that the participation of endosomal epsins and GGAs in this trafficking step is conserved. To get information about the nature of the defect in Cps1 processing, we analyzed the distribution of Ub:GFP-Cps1 in the same strains. In wild-type, most of the fluorescence was present in the lumen of vacuoles (Figs 4c,d and S4a), which in *S. pombe* are smaller and more numerous than in *S. cerevisiae*. Some small very bright fluorescent dots were also observed within the cytoplasm. These dots corresponded to the PVE, based on their lack of colocalization with the vacuole dye Blue 7-amino-4-chloromethylcoumarin (CMAC) and their colocalization with Cherry-FYVE and with Vps27 (Figs 4c, S4b and S5a). The *ent3Δ* and *ent3Δ gga21Δ* strains exhibited a Cps1 distribution similar to that of the wild-type (Fig. S4a), while *gga21Δ gga22Δ*, *ent3Δ gga22Δ* and *ent3Δ gga21Δ gga22Δ* strains showed low-intensity fluorescence in the vacuole and prominent fluorescence at the PVE (Figs 4c,d and S4b). In the *vps27Δ* strain, Cps1 was observed at the PVE, which was more prominent than in the wild-type, and in the vacuole membrane (Figs 4c,d and S4). These results showed that in cells lacking Gga22 and either Gga21 or Ent3, Cps1 was not properly sorted at the PVE, resulting in a reduced delivery to the vacuole lumen.

To complement these results, we analyzed GFP-Cps1 colocalization with Cfr1-RFP in wild-type, *gga21Δ gga22Δ* and *ent3Δ gga22Δ* strains. In the wild-type strain, little colocalization of Cps1 and Cfr1 was observed (Fig. 4e,f). By contrast, substantial colocalization was detected in the *gga21Δ gga22Δ* and *ent3Δ gga22Δ* strains, showing a defect in Cps1 exiting from the TGN in these mutants.

In summary, Gga22/Gga21 and Gga22/Ent3 collaboration was required for efficient Cps1 exiting of the TGN. In addition, the fraction of Cps1 that reached the PVE in the absence of these adaptors was not sorted to the vacuole efficiently, and underwent abnormal processing. Therefore, the same adaptors regulated events of Cps1 trafficking that occurred in different membranous organelles, the TGN and the PVE.

### Vph1 transport to the vacuole is efficient in the absence of the GGA and epsin adaptors

**Figure 5:**
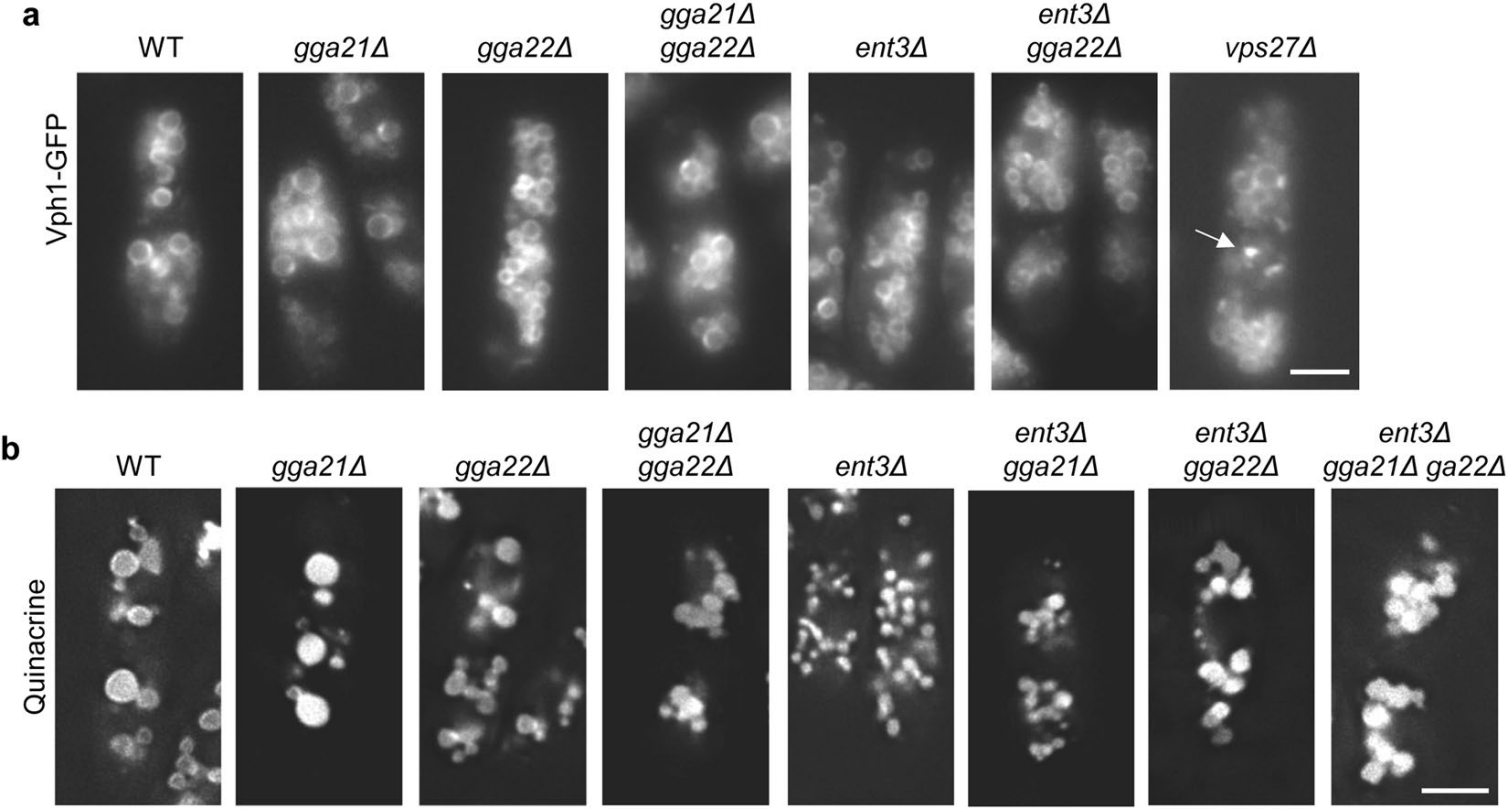
Localization and functionality of the v-ATPase. **(a)** Distribution of the v-ATPase V0 subunit Vph1 in different strains. The images were captured with a Nikon 90i microscope. The arrow denotes an abnormal accumulation of Vph1-GFP in *vps27Δ*. **(b)** Vacuoles from the indicated strains were stained with the acidic vesicle tracer quinacrine. The images are single planes captured with a DeltaVision system. Bar, 5 µm.

The v-type ATPase V0 subunit Vph1 is a multi-pass membrane protein that follows the CPY pathway, and is sorted at the PVE to reach the vacuole membrane^4,45,46^. The analysis of Vph1-GFP localization in the wild-type and in mutants lacking GGA and/or Ent3 adaptors showed that for all strains the protein was distributed along the vacuole membrane (Fig. 5a). In *vps27Δ*, Vph1 was observed along the vacuole membrane and in some intracellular foci, which might correspond to the class E endosome, a structure smaller in *S. pombe* than in *S. cerevisiae*^44^. Quinacrine staining showed that the vacuoles in all the adaptor mutants were acidic, indicating that other ATPase subunits were properly delivered and assembled, producing a functional ATPase (Fig. 5b). These result showed that Gga22/Gga21 and Gga22/Ent3 collaboration did not facilitate the trafficking of all the proteins that follow the CPY pathway.

### A fraction of Vps10 undergoes partial proteolytic processing at the PVE in *gga21Δ gga22Δ*, *ent3Δ gga22Δ* and *ent3Δ gga21Δ gga22Δ* mutants

In *S. pombe*, Vps10 undergoes a partial proteolytic cleavage in all the retromer mutants^8^. Subtle Vps10 instability has also been reported in some *S. cerevisiae* retromer mutants, as data not shown for *vps26Δ* and as a subtle band in the *vps35Δ* blot in Fig. 7, lane 3 h of Cereghino *et al*.^34^; although, in this organism Vps10 cleavage is more evident in ESCRT mutants. Partial Vps10 cleavage happens because its N-terminal end is exposed to the protease Pep4 in an acidic environment when its sorting at the PVE/vacuole interface is inefficient^34,46^. The lack of cleavage in the *gga21Δ gga22Δ vps28Δ* strain was interpreted as a consequence of blocking the exit of Vps10 from the TGN in the absence of the GGA proteins^16^.

**Figure 6:**
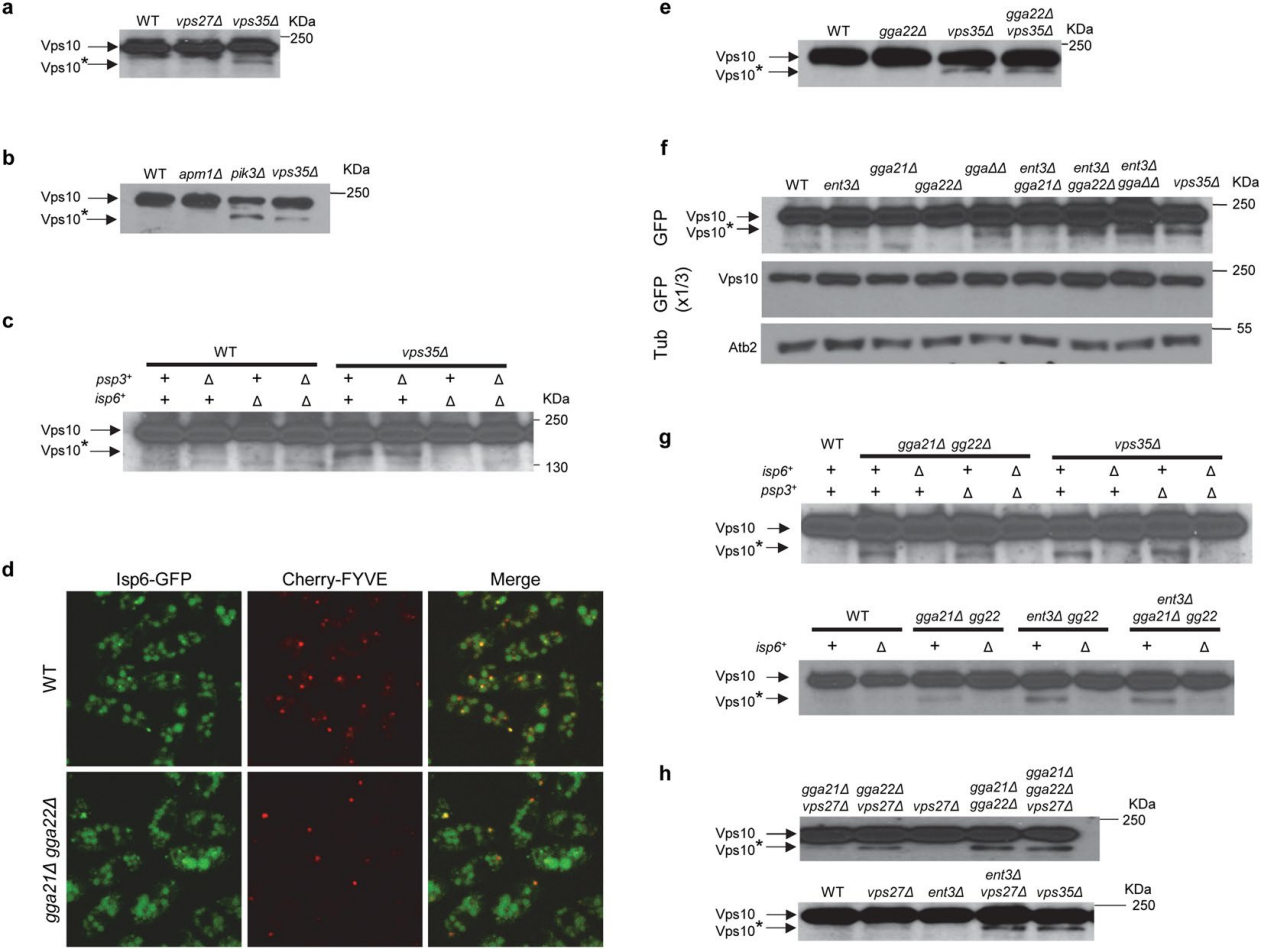
GGA and Ent3 proteins are involved in Vps10 retrograde trafficking. In (**a**–**c**) and (**e**–**h**), equal amounts of lysate from the indicated strains containing Vps10-GFP were subjected to SDS-PAGE and immunoblotted with anti-GFP. Vps10 denotes Vps10-GFP, and Vps10* denotes a Vps10-GFP truncated form. All full-length blots are shown as supplementary material. **(a)** Vps10 is processed in *vps35Δ*. **(b)** Vps10 is processed in *pik3Δ*. **(c)** Isp6 is the protease responsible for Vps10 cleavage in *vps35Δ*. **(d)** Isp6 localizes at the vacuoles and the prevacuolar endosome (labelled with Cherry-FYVE). Images are single planes captured with a spinning-disk confocal microscope. Bar, 10 µm. **(e)** Vps10 is processed in *gga22Δ vps35Δ*. **(f)** Vps10 is processed in *gga21Δ gga22Δ* (*ggaΔΔ*), *ent3Δ gga22Δ* and *ent3Δ gga21Δ gga22Δ* (*ent3Δ ggaΔΔ*). Blots were decorated with anti-GFP (upper and middle planes) and with anti-tubulin (Tub, lower panel; loading control). The middle panel corresponds to a low exposure of the blot shown in the upper panel. **(g)** Isp6 is the protease responsible for Vps10 cleavage in *gga21Δ gga22Δ*, *ent3Δ gga22Δ* and *ent3Δ gga21Δ gga22Δ*. **(h)** Vps10-GFP is processed in *gga22Δ vps27Δ*, *gga21Δ gga22Δ vps27Δ* and in *ent3Δ vps27Δ* strains.

As described above in the *gga21Δ gga22Δ*, *ent3Δ gga22Δ* and *ent3Δ gga21Δ gga22Δ* strains, Vps10 was partially retained at the TGN and partially delivered to the PVE (Figs 1–3). In order to complement these results, and get additional information about the trafficking events where the GGA and Ent3 proteins collaborate, we analyzed Vps10 stability in different strains. First, we compared the electrophoretic mobility of Vps10-GFP from wild-type, *vps35Δ* and *vps27Δ* strains. A fast-mobility band was detected in the retromer mutant, but not in the ESCRT mutant (Fig. 6a). This band had low intensity, indicating that only a small fraction of the Vps10 molecules was subjected to cleavage. To understand the cell environment where this cleavage occurred, we analyzed protein extracts from wild-type, *apm1Δ*, *pik3/vps34Δ* and *vps35Δ* strains. The degradation band was present in extracts from the latter two strains, but not in those of the wild-type and *apm1Δ* strain (Fig. 6b), strongly suggesting that Vps10 cleavage occurred at the PVE/vacuole interface, not at the TGN. Partial Vps10 proteolysis was also reported in the budding yeast *vps34Δ* mutant^35^. There is no Pep4 homologue in fission yeast, and the vacuolar proteases Isp6 and Psp3 are both required for Cpy1 and ALP processing^47^. To confirm that the fast-mobility Vps10 form was produced by proteolysis, *isp6*^+^ and/or *psp3*^+^ genes were deleted in the control and *vps35Δ* strains. Western blotting showed that the degradation band was present in *vps35Δ* and *vps35Δ psp3Δ*, but not in *vps35Δ isp6Δ* and *vps35Δ psp3Δ isp6Δ* extracts (Fig. 6c). These results confirmed that this band was the result of Vps10 proteolysis, and that the protease responsible for this processing was Isp6. Isp6-GFP microscopy showed that this protease was present in both the vacuole and the PVE (Fig. 6d).

When we determined Vps10 cleavage in *gga22Δ vps35Δ* extracts, we found that the electrophoretic pattern in this strain was similar to that observed in the single *vps35Δ* mutant (Fig. 6e), confirming that a fraction of the receptor exited the TGN in the absence of Gga22. We could not get *gga21Δ gga22Δ vps35Δ* or *ent3Δ vps35Δ* strains, showing a strong genetic interaction between *vps35*^+^ and the adaptor genes. This result suggested that GGA and Ent3 proteins might participate in a process related to the function of retromer. To address the possibility that Gga22/Gga21 and Gga22/Ent3 collaboration contributed to the efficiency of the PVE to TGN retrograde trafficking, we analyzed Vps10 cleavage in *vps35*^+^ strains lacking GGA and/or Ent3 proteins. A fast-mobility band similar to that observed in *vps35Δ* was detected in *gga21Δ gga22Δ*, *ent3Δ gga22Δ*, and *ent3Δ gga21Δ gga22Δ* extracts, but not in *gga21Δ*, *gga22Δ*, *ent3Δ* and *ent3Δ gga21Δ* extracts (Fig. 6f). This band was produced by Isp6-dependent proteolysis (Fig. 6g). Isp6 was properly localized in the *gga21Δ gga22Δ* mutant (Fig. 6d) and, based on western blotting, Vps10 cleavage was not due to an increase in the amount of Isp6 in this strain (data not shown). All these results showed that Vps10 processing was similar in *vps35Δ*, *gga21Δ gga22Δ*, *ent3Δ gga22Δ*, and *ent3Δ gga21Δ gga22Δ* strains. Since it took place at the PVE/vacuole interface, and Vps35 was localized at the PVE in the *gga21Δ gga22Δ* and *ent3Δ gga22Δ* strains (Fig. S5), they indicated that Gga22 collaboration with Gga21 and with Ent3 facilitated an efficient Vps10 retrograde trafficking from the PVE to the TGN. Inefficient Vps10 retrograde trafficking in the adaptor mutants was also supported by the fact that a degradation band similar to that produced in *vps35Δ* was observed in *gga22Δ vps27Δ*, *gga21Δ gga22Δ vps27Δ* and *ent3Δ vps27Δ* strains (Fig. 6h). The participation of clathrin adaptors other than AP-1 in Vps10 retrograde transport was consistent with the fact that *S. cerevisiae* Vps10 is unstable in clathrin mutants, but not in *apl2Δ*^35^. In budding yeast experiments directed to analyze Vps10 stability in strains lacking both GGAs indicated that the receptor was stable and, therefore, that these adaptors were not involved in retrograde transport^14,16^. In these experiments, the results were compared with those of ESCRT mutants, with a severe alteration in the PVE. Since, based on our results, the degradation band represents a minor fraction of the total Vps10, its contribution to the protein stability is probably subtle and, therefore, it might have been underappreciated in the pulse-chase analysis performed in *S. cerevisiae*.

The results described in this section showed that the same adaptors facilitated anterograde and retrograde trafficking events of the same cargo for a transport route. Nevertheless, the role of Ent3 and GGAs in Vps10 retrograde transport was less prominent than that in its anterograde transport, and might be indirect.

### The Syb1 SNARE localizes at the PVE in *gga21Δ gga22Δ*, *ent3Δ gga22Δ*, and *ent3Δ gga21Δ gga22Δ* cells

**Figure 7:**
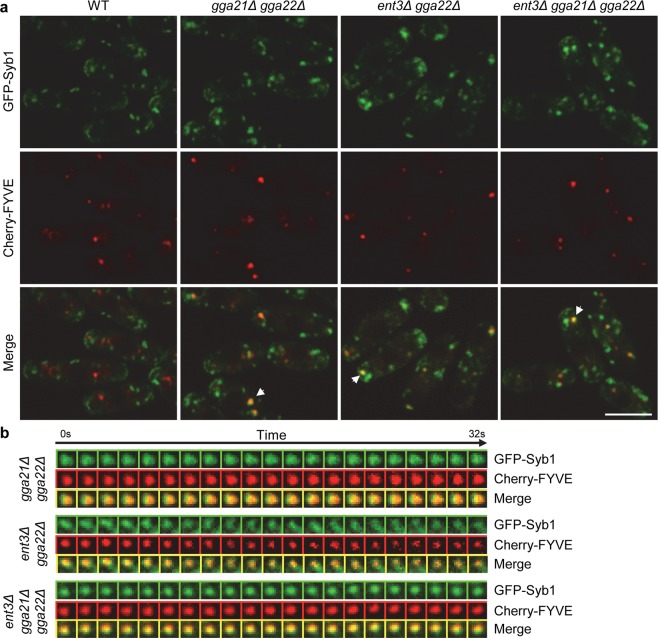
Syb1 localizes in the prevacuolar endosome in *gga21Δ gga22Δ*, *ent3Δ gga22Δ* and *ent3Δ gga21Δ gga22Δ* cells. **(a)** Single-plane images from wild-type (WT) *gga21Δ gga22Δ*, *ent3Δ gga22Δ* and *ent3Δ gga21Δ gga22Δ* cells, containing GFP-Syb1 and Cherry-FYVE, captured with a spinning-disk microscope. Arrowheads denote the PVEs represented in (b). Bar, 10 µm. **(b)** Tile views of PVEs from *gga21Δ gga22Δ*, *ent3Δ gga22Δ* and *ent3Δ gga21Δ gga22Δ* strains containing GFP-Syb1 and Cherry-FYVE. Photobleach-corrected images are single planes, from representative time-lapses obtained imaging the cells at 0.81 second-intervals; every other time-point is represented. The arrow represents time progression from the initial image (Time 0 s).

Syb1/Snc1 is an exocytic SNARE that cycles between the plasma membrane and the TGN. This recycling is defective in *S. pombe gga21Δ gga22Δ* mutant^30^, and in *S. cerevisiae gga1Δ gga2Δ*, *ent3-1* and *ent3Δ ent5Δ* mutants^48,49^. In all these strains, Syb1/Snc1 remains largely intracellular; however, the nature of the compartment where the SNARE localizes has not been determined. We investigated the distribution of Syb1 in *gga21Δ gga22Δ*, *gga22Δ ent3Δ* and *gga21Δ gga22Δ ent3Δ* cells expressing GFP-Syb1 and Cherry-FYVE. In wild-type, there was no GFP/Cherry colocalization (Fig. 7a). However, in *gga21Δ gga22Δ*, *gga22Δ ent3Δ* and *ent3Δ gga21Δ gga22Δ* strains the proteins colocalized in some intracellular dots. Analysis of fluorescence along time showed that both proteins coexisted in these dots for a minimum of 30 seconds (Fig. 7b). These results showed that Syb1 localized in the PVE stably when both GGAs or Gga22 and Ent3 were absent. The fact that Syb1/Snc1 recycling depends on the sorting nexin Atg24/Snx4^50^, which localizes in the same membranes as the retromer^51^, indicates that it undergoes recycling through the PVE. Our results were consistent with this hypothesis and suggested that this recycling was considerably slower or less efficient in the absence of GGA and Ent3 adaptors. These results also showed that Gga22 collaborated with Gga21 and with Ent3 in the trafficking of a protein that does not follow the CPY pathway.

### Correct organization of the PVE requires GGAs and Ent3

**Figure 8:**
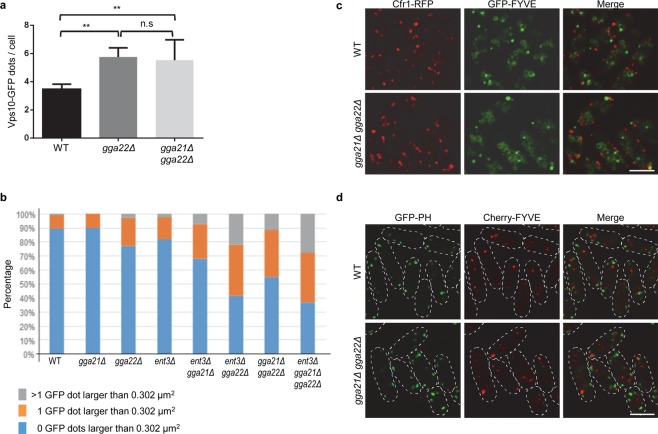
Characteristics of prevacuolar endosomes in adaptor mutants. **(a)** Number of Vps10-GFP dots per cell in wild-type (WT), *gga22Δ* and *gga21Δ gga22Δ* cells containing Vps10-GFP. ImageJ software was used to score fluorescent dots ≥ 0.071 µm^2^ from single-plane images. Cells from six independent experiments were analyzed. The mean, standard deviation and statistical significance, determined by Tukey’s multiple comparisons test after ANOVA, is shown. **p < 0.01; ns, non-significant. **(b)** Percentage of cells from the indicated strains with enlarged PVCs. ImageJ software was used to score GFP dots larger than 0.302 μm^2^ from sum projection-images captured with a confocal spinning-disk microscope. A minimum of 130 cells from a minimum of two experiments were scored. The mean is represented for each value. **(c)** Distribution of Cfr1-RFP and GFP-FYVE in WT and *gga21Δ gga22Δ* cells. **(d)** Distribution of GFP-PH and Cherry-FYVE in WT and *gga21Δ gga22Δ* cells. The dashed lines denote the cell periphery. Bar, 10 µm.

When we analyzed the localization of Vps10-GFP, we observed that the sizes of the fluorescent dots were more heterogeneous in the *gga22Δ* and *gga21Δ gga22Δ* mutants than in the wild-type (Fig. 1e,g). The mutants exhibited numerous tiny fluorescent dots. Additionally, the number of dots with a minimum size similar to that of the GFP dots observed in the control strain (0.071 µm^2^) was greater in the mutants (Fig. 8a). Furthermore, over 45% of the *gga21Δ gga22Δ* cells exhibited one or more dots larger than 0.301 µm^2^ (Fig. 8b). According to their colocalization with Cherry-FYVE, these dots were enlarged PVEs (arrowheads in Fig. 1e). Large fluorescent dots were also observed in this mutant using other PVE markers, Vps27-RFP, Nhx1-GFP, GFP-Pep12, and Vps35-GFP (Fig. S5). These results were in agreement with the abnormal organization of the PVE in this mutant observed by electron microscopy^30^. The *ent3Δ gga22Δ* and *ent3Δ gga21Δ gga22Δ* cells also exhibited enlarged PVEs (Figs 3b, 8b and S5). These results demonstrated that Gga22/Gga21 and Gga22/Ent3 collaborations were required for correct PVE organization.

*S. cerevisiae vps* mutants lacking the ESCRT exhibit an aberrant PVE (the class E endosome^52^), which contains PVE/vacuole markers as well as TGN markers (GGA adaptors, Ent5, Vps10, Cpy1 and Vph1^17,27,34,46,52–54^). In the *S. pombe gga21Δ gga22Δ* strain there was no FYVE/Cpy1 colocalization (Fig. 2a). Additionally, Vph1 localization was similar in the wild-type, *gga21Δ gga22Δ* and *ent3Δ gga22Δ* strains, while its distribution was slightly altered in the *vps27Δ* strain (Fig. 5a). Furthermore, there was no colocalization between FYVE and the TGN markers Cfr1 and hFAPP1(PH) in either the wild-type or the *gga21Δ gga22Δ* strains (Fig. 8c,d). These results, together with the fact that Cps1 localization and processing, and Vps10 cleavage were different in the adaptor and the *vps27Δ* mutants (Figs 4 and 6), showed that *gga21Δ gga22Δ* and *ent3Δ gga22Δ* mutants were not class E *vps* mutants. A similar conclusion was reached for the budding yeast *gga1Δ gga2Δ* mutant^14–16^. Therefore, although the aberrant structures generated in the absence of the adaptors and the ESCRT contain Vps10, their nature was different.

### Growth under stress conditions is compromised in mutants lacking GGA, Ent3 and ESCRT components

**Figure 9:**
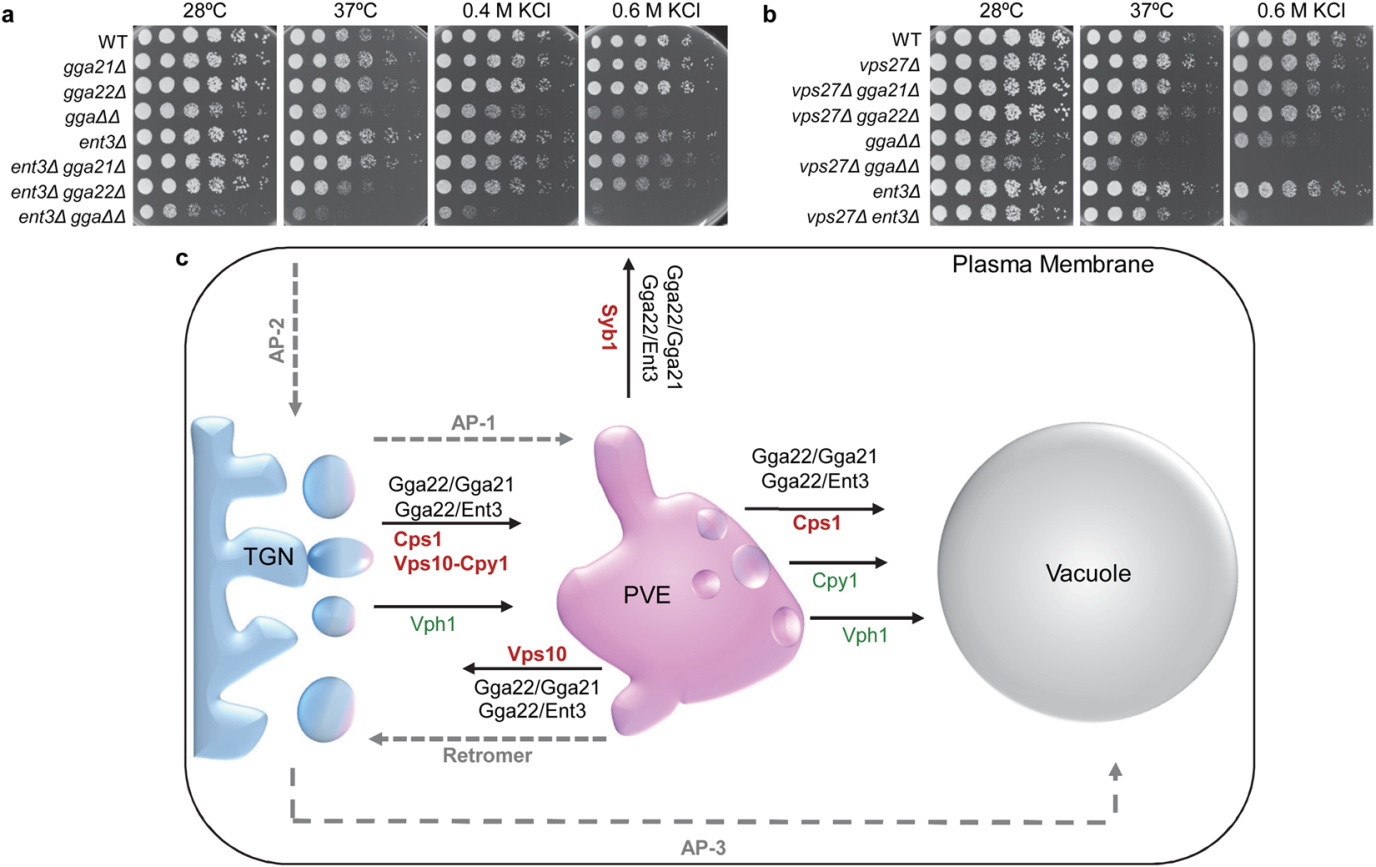
Gga22 and Ent3 participate in multiple trafficking events required for efficient growth under stress. **(a)** Genetic interaction between *gga21Δ*, *gga22Δ* and *ent3Δ*. **(b)** Genetic interaction between *vps27Δ*, *gga21Δ*, *gga22Δ* and *ent3Δ*. In (**a**,**b**), 3 × 10^4^ cells and serial 1:4 dilutions from the indicated strains were spotted on YES plates and incubated at the indicated temperatures for three days. **(c)** Trafficking pathways where Gga22 collaborates with Gga21 and with Ent3. For each pathway, the proteins whose trafficking is less efficient in *gga21Δ gga22Δ* and in *ent3Δ gga22Δ* mutants are written in bold red lettering while unaffected proteins are written in plain green lettering. Other protein complexes that participate in protein transport between the TGN, the PVE, the vacuole and the plasma membrane are written in grey lettering, and grey dashed lines denote their trafficking routes.

The results described in this study showed that Gga22 collaborates with Gga21 and Ent3 in multiple trafficking steps. To understand whether this collaboration was relevant for cell physiology, we analyzed genetic interactions by assessing growth of different strains at 37 °C (thermal stress) and in the presence of KCl (osmotic and saline stress). The *gga21Δ gga22Δ* and *ent3Δ gga22Δ* strains exhibited reduced growth at 37 °C, while the *ent3Δ gga21Δ gga22Δ* mutant had the strongest growth defect (Fig. 9a). Additionally, the *gga21Δ gga22Δ* strain exhibited reduced growth in the presence of KCl, a defect that was enhanced by the *ent3Δ* deletion (Fig. 9a). As mentioned above, we could not get strains *vps35Δ gga21Δ gga22Δ* and *vps35Δ ent3Δ*, showing genetic interactions between the adaptor and retromer mutants. We analyzed interactions between adaptor and ESCRT mutants, defective in protein sorting at the PVE. The *vps27Δ* mutation reduced the growth of the *gga21Δ gga22Δ* and the *ent3Δ* cells at 37 °C, and in the presence of KCl (Fig. 9b). Therefore, although eliminating the GGA and epsin adaptors did not completely block any of the trafficking steps where they participated, their absence was relevant under stress conditions, suggesting that these adaptors act as a fine-tuning mechanism to ensure cell survival under stress. The fact that many of the phenotypes described in this work for *gga21Δ gga22Δ* and *ent3Δ gga22Δ* mutants were similar, suggested that Gga21 and Ent3 acted in the same functional pathway. However, the different growth of *vps27Δ gga21Δ* and *vps27Δ ent3Δ* cells under stress (Fig. 9b) argued against this hypothesis, consistent with the enhanced phenotypes of the triple *ent3Δ gga21Δ gga22Δ* mutant. These results showed that the functional relationship between the GGA and Ent3 adaptors was complex.

## Discussion

Protein trafficking between the Golgi and the lysosome/vacuole is a complex process that is not well characterized. Analysis of a few model cargoes, including some chimeras, has helped to determine the participation of vesicle coats and adaptors in certain steps of the different trafficking routes between these organelles^4,6,7,55,56^ (Fig. 9c). While initial models suggested that an adaptor would only participate in anterograde or retrograde trafficking of certain cargoes, later studies have demonstrated that protein trafficking is complex, and that the involvement of multiple adaptors is a hallmark of clathrin-dependent trafficking. Thus, physical and functional interactions between AP-1 and Gga2, between AP-1 and endosomal epsins, and between Gga2 and the endosomal epsins have been described^16–22,24,25,27,57,58^. The current study extended these results and demonstrated that the GGAs and the endosomal epsin Ent3 collaborate in multiple steps of Vps10 and Cps1 trafficking, as had previously been suggested^24^. Participation of AP-1, the GGAs and the endosomal epsins in Vps10 sorting at the TGN had been widely analyzed^16–19,21,22^; however, direct analysis of the involvement of the GGAs and Ent3 in Cps1 exiting of the TGN had not previously been reported. Our results showed that in the absence of these adaptors, both cargoes exit the TGN less efficiently.

Nevertheless, a fraction of the Vps10 and Cps1 proteins reached the PVE in the *gga21Δ gga22Δ*, *ent3Δ gga22Δ*, and *ent3Δ gga21Δ gga22Δ* mutants. Furthermore, a *S. cerevisiae* Vps10 protein lacking its cytoplasmic C-terminus is sorted to the PVE, and clathrin itself is not essential for Vps10 exiting the TGN^34,35,40,59,60^. These results demonstrate that trafficking of these proteins is flexible regarding their requirements, and are consistent with the hypothesis that part of anterograde Cpy1 trafficking is receptor-aided, while another part happens by bulk flow protein trafficking^4,8,61^. It is possible that endosome maturation participates in such bulk flow membrane protein trafficking. The fact that Vph1, a cargo that populates the CPY pathway^4,45,46^, is not retained in the TGN in *gga21Δ gga22Δ*, *ent3Δ gga22Δ*, and *ent3Δ gga21Δ gga22Δ* strains might be explained by the nature of this protein, at least partially. While Vps10 and Cps1 are single-pass membrane proteins, Vph1 is polytopic. It is known that the length and composition of transmembrane domains influences protein localization^48,62,63^. It is possible that single-pass proteins are dependent on adaptors to be stabilized at the vesicle budding site and/or to be efficiently sorted into clathrin-coated vesicles, while polytopic proteins, which tend to associate, are more stable at these sites and do not need the adaptors to be sorted, or are more prone to bulk flow trafficking. We cannot rule out the possibility that the simultaneous elimination of AP-1, the GGAs, Ent3, and maybe other adaptors, would block the exit of Cps1 and Vps10 from the TGN, which would agree with the severe growth defects produced by the simultaneous deletion of AP-1 and GGA genes^16^. Nevertheless, this strong genetic interaction could also be explained by limitations in the trafficking of multiple cargoes.

The GGAs and Ent3 have been shown to be involved in protein sorting into the PVE intraluminal vesicles^27,28,57^. Our results showed that both types of adaptors collaborated for efficient cargo sorting at the PVE. Thus, the Cps1 molecules that exit the TGN suffered a partial retention at the PVE and/or underwent a slow delivery to the vacuole, resulting in an abnormal processing. Nevertheless, Cps1 was not redistributed to the vacuole membrane, showing that the defect in its sorting was different from, and probably occurred before that in the ESCRT mutants^42,44,64^. In the wild-type, the Vps10-Cpy1 complex disassembles at the PVE and the receptor undergoes retrograde trafficking to the TGN in a retromer-dependent process^8,9^. In the adaptor mutants, a small fraction of the Vps10 molecules that reached the PVE was subjected to partial proteolytic degradation, probably because it was retained at this organelle or recycled back to the TGN slowly and consequently exposed to Isp6 for longer than in the control. Based on genetic interactions, this inefficient retrograde transport would result from a defect in a process parallel to that mediated by the retromer. The equilibrium between i) continuous arrival of a few Vps10 molecules from the TGN to the PVE; ii) continuous recycling of most of these few molecules to the TGN; and iii) partial cleavage of the rest of the molecules, would explain why Vps10 did not accumulate at the PVE even though retrograde trafficking was inefficient. With regard to Cpy1, a fraction of this protein was found in the vacuole lumen of mutant cells; probably, these molecules were released into the PVE and rapidly transferred to the vacuole after both organelles fuse or undergo “kiss-and-run” events, as proposed recently^2^. Another Cpy1 fraction was missorted to the cell surface because of the partial defect in Vps10 trafficking. The collaboration between the GGA and epsin adaptors in a function at the PVE was in agreement with GGAs being detected at this organelle under certain conditions^17,53^, and Ent3 binding phosphatidylinositol-3,5-bisphosphate (PI(3,5)P2) and participating in protein sorting into the PVE^27,28^. Contrary to other cargoes of the CPY pathway, Vph1 was not retained at the PVE in the adaptor mutants. This difference might be explained by its polytopic nature and/or because this protein does not need to be sorted back to the TGN or into the PVE even in the wild-type. Thus, once in the endosomal membrane Vph1 might reach the vacuole by bulk flow trafficking, in agreement with the fact that its sorting is not altered in the absence of PI(3,5)P2^28^.

Our results showed that the GGA and Ent3 proteins played a role in maintaining PVE organization. This role might be exerted through a function that might be additional to their function as cargo adaptors for the CPY pathway, since they collaborated in multiple trafficking events. For example, they might help to organize microdomains in the organelle membrane that modulate its fluidity/flexibility and fusion/fission capacity, or they might help to recruit proteins that influence these aspects of the membranes. Another possibility could be that the adaptors themselves play a transitory structural role at the vesicle budding site. The absence of this function would alter the trafficking of each cargo to a different extent. Adaptor mutants do not exhibit a general trafficking defect. Thus, Pep12, Vps10 and Cps1p trafficking was not affected by mutations in AP-1 alone^24^ (Fig. 1). Furthermore, Vph1 was not retained at the PVE in the *gga21Δ gga22Δ* and *ent3Δ gga22Δ* mutants (Fig. 5). Nevertheless, absence of clathrin adaptors might alter the input/output balance of proteins and lipids in endomembranes, leading to alterations in these organelles. The abnormal Cps1 trafficking and processing, and the reduced Vps10 and Syb1 recycling showed that the functionality of the PVE was defective in *gga21Δ gga22Δ*, *ent3Δ gga22Δ*, and *ent3Δ gga21Δ gga22Δ* cells. We cannot rule out the possibility that these alterations in cargo trafficking were an indirect consequence of the defects in the organization of the PVE produced in the mutants. In any case, our results showed that the adaptors facilitated these trafficking events, which were inefficient in the mutants.

In this work we showed collaboration between the GGAs and Ent3, in particular between the dominant Gga22 and either Gga21 or Ent3, in regulating multiple aspects of cargo trafficking. What is the nature of this collaboration? The simplest answer is that these adaptors play cooperative mechanical roles within a single trafficking event, as happens with proteins that regulate endocytosis^65^, or cross-regulatory events, as shown for AP-1, Gga2 and budding yeast endosomal epsins^20,58^, and for AP-1 and EpsinR in Metazoa^66^. It is possible that each of the adaptors contributes to stabilize the interaction between the cargoes or clathrin with another adaptor. Indeed, reduced Pep12-Gga2 and reduced clathrin-Gga2 interactions have been described in *S. cerevisiae* epsin mutants^24^.

The genetic and functional interactions between the GGAs, between Ent3 and the GGAs, and between these adaptors and Vps27 might be related to their capacity to bind ubiquitinated proteins^27,67^. This would agree with the fact that Cps1 is ubiquitinated. However, various screening and prediction analyses^68,69^ indicate that Vph1 is ubiquitinated, while Vps10 is not, arguing against this hypothesis. Additionally, we detected Gga22/Gga21 and Gga22/Ent3 collaboration at both the TGN and the PVE, while the ubiquitin-binding function of Gga2 is required for protein sorting into the PVE but not at the TGN^57,70^.

Epsins act as adaptors for some SNAREs, as Vti1, Pep12 and Syn8^49,66,71^. Pep12 is a CPY pathway cargo that undergoes abnormal trafficking in the *S. cerevisiae gga1Δ gga2Δ* mutant, where it cofractionates with the TGN marker Tlg1^48^. Therefore, the GGAs and Ent3 proteins might collaborate in regulating this SNARE, whose defective trafficking might in turn alter some aspects of Vps10, Cps1, and Syb1/Snc1 trafficking. Nevertheless, in the *S. pombe* adaptor mutants the pattern of Pep12 localization was similar to that of FYVE^30^, which did not colocalize with TGN markers (Fig. 8). The different Pep12 behavior might reside in the fact that in *S. cerevisiae* the experiments were performed using chimeras, which might not behave exactly like the native protein. Additionally, Pep12 function is exerted at the PVE^72,73^, and we detect Gga22/Gga21 and Gga22/Ent3 collaboration both in the TGN and in the PVE. Therefore, although a defect in Pep12 trafficking in the adaptor mutants cannot be rule out, this defect by itself did not seem to be responsible for all the abnormalities detected in *gga21Δ gga22Δ*, *ent3Δ gga22Δ*, and *ent3Δ gga21Δ gga22Δ* mutants.

Previous results showed that the GGA proteins participate in the trafficking of certain cargoes along specific routes. Thus, they facilitate anterograde trafficking of Vps10 and mannose-6-phosphate receptors from the TGN to the vacuole, Chs3, Snc1, and BACE1 retrograde intra-TGN trafficking, LRP9 retention at the TGN, and GLUT4 delivery to a specialized insulin-responsive compartment^24,38,48,74–78^. Our results showed that efficient anterograde and retrograde trafficking events of the same cargo (Vps10) along the same route requires the cooperation between the GGAs and Ent3. In addition, the participation of AP-1 in both anterograde and retrograde transport events at the TGN has been reported^19,75,79,80^. Therefore, the participation of clathrin adaptors in both directions of the same trafficking pathway seems to be more frequent than initially suspected.

Alteration in the homeostasis of the endosomal-lysosomal system is related to human disease, in particular to neurological disorders^1^. This homeostasis requires adequate protein transport to the lysosome, a process that is not well understood. Increasing the knowledge about this transport will help to understand the nature of those disorders. This work extended the previous knowledge and showed that monomeric clathrin adaptors regulate multiple trafficking events to and from the prevacuolar endosome (Fig. 9c).

## Methods

All materials, data and associated protocols used in this work are available upon request.

### Strains and growth conditions

All general growth conditions and yeast manipulations were as previously described^81,82^. The relevant genotypes and the source of the strains used are listed in Table S1. Unless otherwise stated, the experiments were performed with cells growing exponentially in liquid rich medium, yeast extract with supplements (YES; 0.5% yeast extract, 3% glucose, 225 mg/l adenine sulphate, histidine, leucine, uracil and lysine, 2% agar), and incubated at 28 °C. Geneticin, hygromycin and nourseothricin were used at 120 μg/ml, 400 µg/ml, and 50 μg/ml, respectively. Latrunculin A (stock at 5 mM in DMSO) was used at 100 μM for 10 minutes.

### Genetic methods

Molecular and genetic manipulations were according to Sambrook *et al*.^83^. Gene deletions and C-terminally tagged proteins were generated by transforming *pku70Δ* strains^84^ with polymerase chain reaction (PCR)-generated modules, as described^85^. The resulting transformants were backcrossed to reintroduce the *pku70*^+^ allele. The pleckstrin homology (PH) domain, used as a phosphatidylinositol-4-phosphate(PI4P)-binding probe, was PCR-amplified from plasmid pRS406-PHO5-GFP-hFAPP1(PH) domain^86^ (#58723 Addgene) as a *Mlu*I/*Sal*I DNA fragment. It was ligated to the C-terminal end of green fluorescent protein (GFP) or mCherry, a red fluorescent protein, amplified as *Apa*I/*Mlu*I fragments, and cloned under the control of the *nda2*^+^ 5′ untranslated region (a 571 base pair *Pst*I/*Apa*I DNA fragment) and 3′ untranslated region (a 313 base pair *Sal*I/*Sac*I DNA fragment) into the pINTH81 vector^84^, digested with *Pst*I and *Sac*I. The plasmid was linearized with *Not*I and integrated into the artificial *hph.171* *K* locus^84^ (Yeast Genetic Resource Center, YGRC #FY23692). The phosphatidylinositol-3-phosphate (PI3P)-binding probe Fab1, YOTB/ZK632.12, Vac1, and EEA1 (FYVE) domain from EEA1^30^ (a *Mlu*I/*Sal*I DNA fragment), was fused to the C-terminal end of GFP or mCherry, and expressed under the control of the *nda2*^+^ promoter and terminator. Depending on the experimental requirements, these constructs were either cloned into pINTH81, as explained above, and integrated into the *hph.171* *K* locus, or cloned into pIJK148 (as a *Pst*I/*Sac*I fragment), linearized with *Tth*111I, and integrated into the *leu1*^+^ locus. The Ub:GFP-Cps1 construct was produced by cloning the following PCR-amplified *S. pombe* DNA fragments into pINTH81, under the control of the *nda2*^+^ promoter and the *nmt1*^+^ terminator: first 228 nucleotides from *ubi4*^+^ (*Apa*I/*Hin*dIII fragment), GFP (*Hin*dIII/*Pst*I fragment), and the *cps1*^+^ open reading frame (*Pst*I/*Sal*I fragment). The plasmid was linearized with *Not*I and integrated into the *hph.171* *K locus*. The accuracy of the constructions and integrations were assessed by DNA sequencing and by PCR, respectively. Genetic crosses and selection of the characters of interest by random spore analysis^81^ were used to combine different traits.

### Protein methods

Tri-chloroacetic acid (TCA) protein precipitation from cell extracts and western blot analysis were performed as described^87^. Cells growing exponentially in 30 ml of YES were collected by centrifugation (900 × *g*), washed with 1 ml cold 20% TCA and resuspended in 50 µl of the same solution. Five hundred µl glass beads were added and the cells were broken in a cold Fast Prep FP120 using three 16-second pulses (speed 6), with 5-minute incubations on ice between pulses. Four hundred µl cold 5% TCA was added to the tube, which was vortexed to wash the beads. Cell extracts were transferred to a clean tube and centrifuged for 10 minutes at 4 °C. The pellets were resuspended in 2% sodium dodecyl sulfate (SDS)/0.3 M Tris base. Protein concentration was determined using Bradford protein assay reagent (Bio-Rad). Samples were equalized with respect to protein content, and boiled in the presence of Laemmli sample buffer (50 mM Tris-HCl, pH 6.8; 1% SDS; 143 mM β-mercaptoethanol; 10% glycerol) for five minutes. Samples were subjected to polyacrylamide gel electrophoresis (PAGE), transferred to PVDF membranes, and incubated in blocking buffer (5% Nestlé non-fat dried milk in TBST: 0.25% Tris, pH 6.8; 0.9% NaCl; 0.25% Tween 20) for 1 hour. Primary antibodies were anti-GFP (JL8, Living Colors; 1∶3000) and anti-α tubulin (clone B-5-1-2; 1∶10000). The secondary antibody was horseradish peroxidase-conjugated anti-mouse (1∶10000). Chemoluminiscent signal was detected on X-ray films using the Western Bright ECL detection kit (Advansta). Colony dot-blots were performed as described^30^. Exponentially-growing cells (6 × 10^4^) were spotted onto a nitrocellulose filter deposited on an Edinburgh minimal medium (EMM) plate^81^ supplemented with 225 mg/l adenine sulphate, histidine, leucine, uracil and lysine. After 4 days of incubation at 28 °C, the filter was washed extensively with TBST to eliminate the cells, and blocked with 1% bovine serum albumin in TBST for 1 hour. The filter was decorated with mouse anti-Cpy1 (anti-*S. cerevisiae* Cpy1; Clone 10A5B5; ABCAM, 1∶150) overnight at 4 °C, and then with a secondary antibody for 90 minutes at room temperature. The blot was developed as described above.

### Vacuole staining

The vacuole lumen was stained with CellView Blue CMAC^88^. One ml cells growing exponentially in YES was washed twice in EMM with supplements^81^, and resuspended in 500 μl of the same medium containing the dye (100 μM final concentration from a 10 mM stock in DMSO). The samples were incubated for 30 minutes in the dark in a tube rotator at room temperature, and then centrifuged and resuspended in 1 ml EMM. They were incubated for an additional 10 minute-period as before, collected by centrifugation, resuspended in a small volume of the same medium, and observed. To assess vacuolar acidification, cells were stained with quinacrine. One ml of cells growing exponentially in YES was collected by centrifugation and resuspended in 0.5 ml yeast extract-peptone-dextrose medium (YEPD) buffered with 50 mM Na_2_HPO_4_ to pH 7.6. Quinacrine was added at 200 µM (from a 20 mM stock in water) and the cells were incubated in the dark for 5 minutes. The samples were centrifuged and the cells were washed with 0.5 ml 2% glucose buffered with 50 mM Na_2_HPO_4_ to pH 7.6, resuspended in a small volume of the same solution and observed.

### Microscopy image acquisition

The following microscopes were used in this study: a Leica DM RXA conventional fluorescence microscope (63x objective; numerical aperture 1.4), equipped with a Photometrics Sensys CCD camera, images were captured using Qfish 2.3 software; a Nikon 90i epifluorescence microscope (100× objective, numerical aperture 1.45) with a Hamamatsu ORCA ER digital camera, Metamorph software was used to capture the images; and an Olympus IX71 microscope (100× objective, numerical aperture 1.4) equipped with a personal DeltaVision system and a Photometrics CoolSnap HQ2 monochrome camera; unless otherwise stated, stacks of 3 Z-series sections corresponding to the cell middle were acquired at 0.2-μm intervals and images were processed using deconvolution Softworx DV software (Applied Precision). For confocal live-cell imaging a spinning-disk Olympus IX-81 microscope was used, equipped with a confocal CSUX1-A1 module (Yokogawa) and an Evolve (Photometrics) camera, images were acquired using Metamorph software and were processed with IMAGEJ software (National Institutes of Health). For colocalization analysis, stacks of three 0.25 µm Z-sections of the cell middle were acquired, and the central plane of each stack was analyzed. To analyze the size of Vps10-GFP dots, stacks of thirteen 0.32 µm Z-sections were acquired, while for time-lapse analysis, images of three 0.25 µm Z-sections from the cell middle were captured at 0.81-second intervals for 33 seconds, unless otherwise stated.

### Image quantification and statistical analysis

The photographs captured by the confocal spinning-disk microscope were saved as 16-bit images. For colocalization analysis, the images were filtered with ImageJ and quantified using the JACoP plugin^89^, adjusting the threshold for each channel and using an object-based method. Dots with an area ≥ 4 pixels (0.071 µm^2^) were scored. For the analysis of Ub:GFP-Cps1/Cfr1-RFP colocalization, images were first filtered, and then the fluorescence of the blue channel (produced by blue CMAC vacuolar staining) was subtracted from the green channel to avoid the signal from vacuoles. The resulting green channel images were analyzed for colocalization with the red channel images using the JACoP plugin, as described above. To analyze for the presence of enlarged PVEs, the Vps10-GFP images were filtered and a sum projection was generated from all the planes in the stack. The threshold was adjusted from the projection, and fluorescent dots with an area larger than 17 pixels (0.302 μm^2^) were scored. All the results were represented and statistically analyzed with Graphpad Prism. The test used in each analysis is specified in the corresponding figure legend.

## Supplementary information

Supplmentary information
