# Supplementary material for: Ent3 and GGA adaptors facilitate diverse anterograde and retrograde trafficking events to and from the prevacuolar endosome
Source: Sci Rep. 2019 Jul 24;9:10747. doi: 10.1038/s41598-019-47035-5 (PMC6656748; doi:10.1038/s41598-019-47035-5)

Ent3 and GGA adaptors facilitate diverse anterograde and retrograde trafficking events to and from the prevacuolar endosome

Francisco Yanguas, Esteban Moscoso-Romero and M.-Henar Valdivieso

Fig. S1 (related to Fig. 1) shows that Cfr1 is a stable TGN marker (a) and illustrates the colocalization between Vps10-GFP and Cherry FYVE (b) and the colocalization between Vps10-GFP and Cfr1-RFP (b, c) in the control strain, single and double mutants lacking GGA adaptors.

Fig. S2 (related to Fig 3) shows the colocalization between Gga22-RFP and GFP-PH, and the colocalization between Ent3-GFP and either Cherry-PH or Cherry-FYVE.

Fig. S3 (related to Fig. 3) shows the localization of Clc1, Apm1, Gga22 and GFP-Pep12 in the wild-type and *ent3Δ* strains.

Fig. S4 (related to Fig. 4) shows the localization of Ub:GFP-Cps1 (a) and its colocalization with Cherry-FYVE (b) in different strains.

Fig S5 (related to Fig. 4 and 8) shows the distribution of several proteins that allow the visualization of enlarged PVEs.

Table S1. List of strains used in this work.

Full-length gels (related to Fig 6)

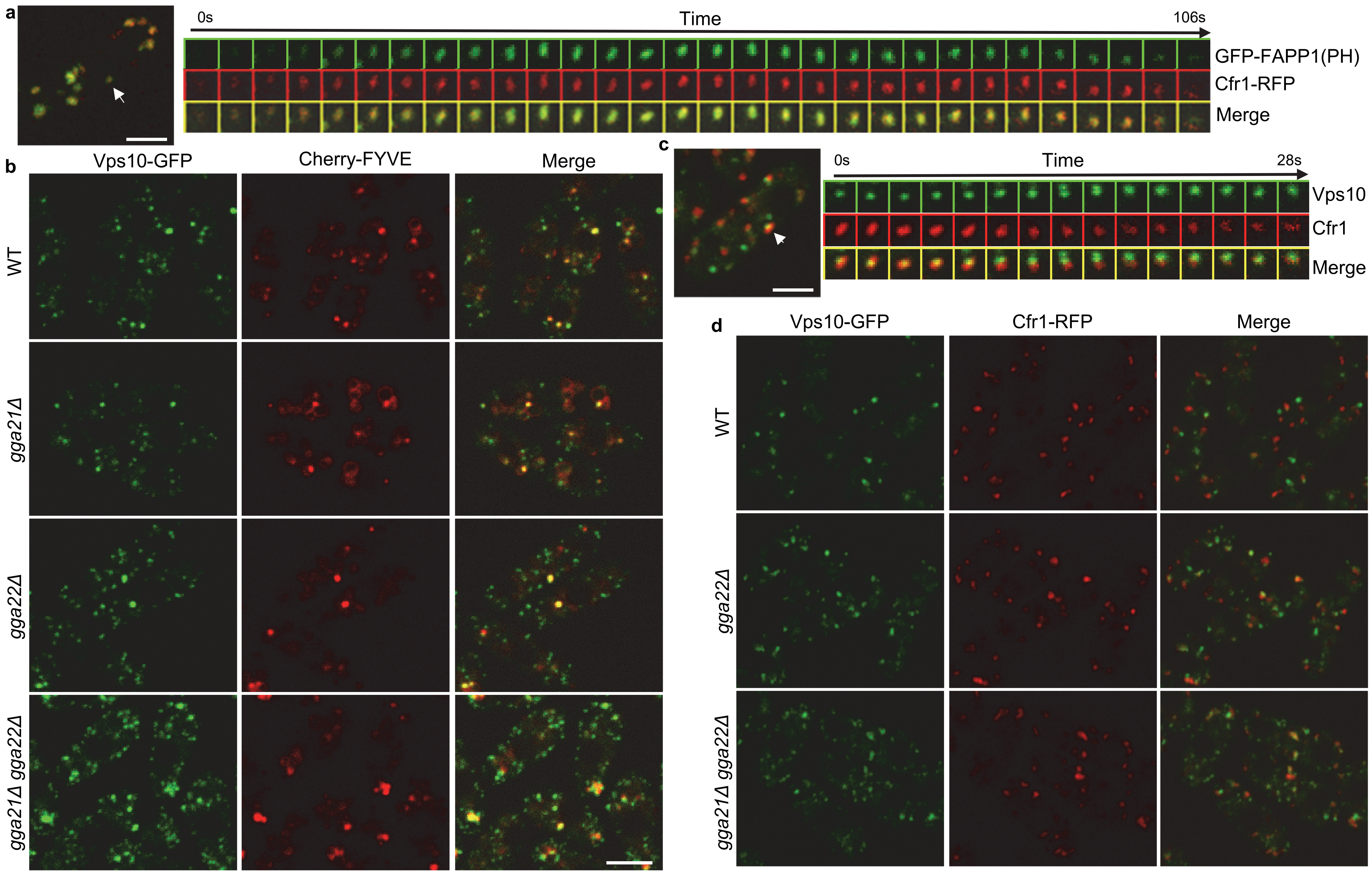

Figure S1. Vps10 distribution. (a) Cfr1-RFP is a stable TGN marker. Cfr1-RFP colocaization with the PI4P probe GFP-hFAPP1(PH) along time is shown. Both fluorescent signals appeared at the same time, coexisted for more than 1.5 minutes, and declined at the same time. Images were captured at 3.52 second-intervals. Eleven whole-cell Z-planes (at 0.38 μm-intervals) were captured. **(b)** Vps10-GFP colocalization with Cherry-FYVE. **(c)** Cfr1-RFP colocalization with Vps10-GFP is dynamic. The fluorescence from both markers was observed together for approximately 10 seconds and later was observed apart. Images were captured at 1.86 second-intervals. Three Z-planes (at 0.3 μm-intervals) from the cell middle were captured. **(d)** Vps10 colocalization with Cfr1-RFP. In (a) and (c) photobleach-corrected images are maximal projection. The arrows denote the particle tracked along time and represented in the tile views. In (b) and (d) images are single planes. In all cases, images were captured with a confocal spinning-disk microscope. Bar, 10 μm.

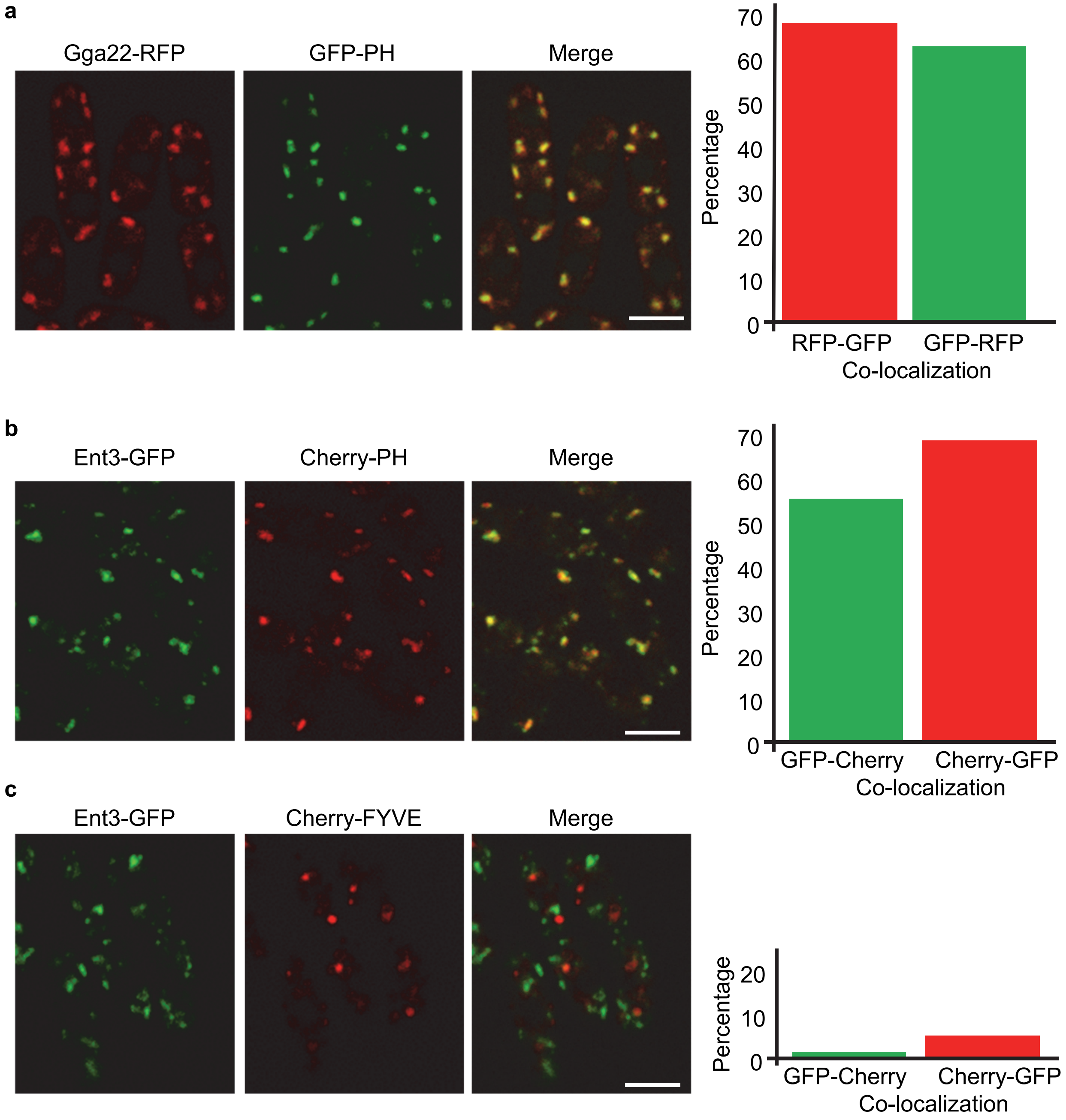

Figure S2. Distribution of Gga22 and Ent3. (a) Gga22 colocalization with the TGN marker GFP-PH. **(b)** Ent3 colocalization with Cherry-PH. **(c)** Ent3 colocalization with the PVE marker Cherry-FYVE. Images are medial planes captured with a confocal spinning-disk microsocope. Bar, 10 µm. For quantification, a minimum of 300 dots were scored for each marker.

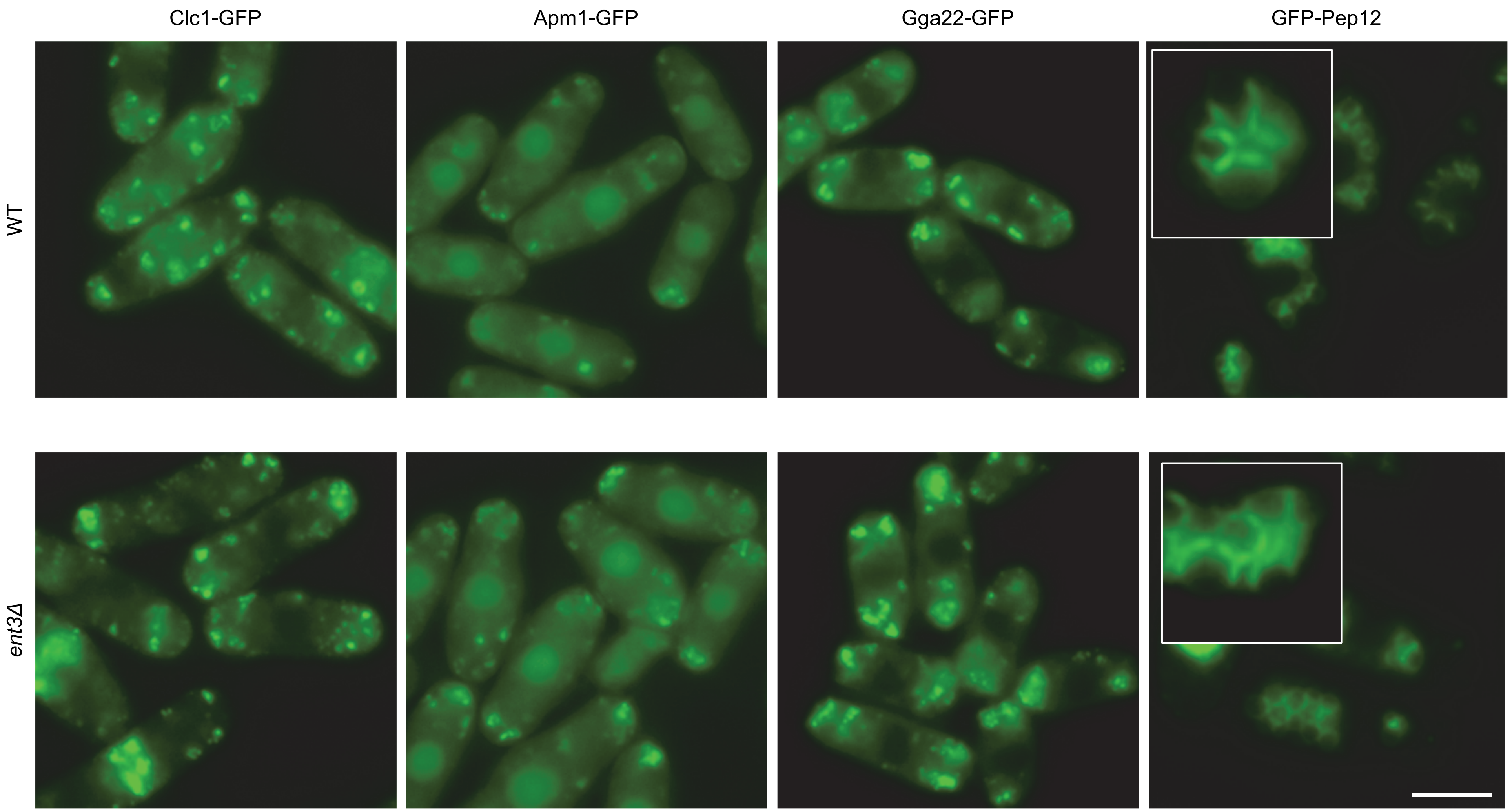

Figure S3. Distribution of proteins involved in vesicle transport in the wild-type and *ent3Δ* strains. Images were captured with a Leica conventional fluorescence microscope. For GFP-Pep12, the insets are enlargements of vacuole clusterss. Bar, 10 µm.

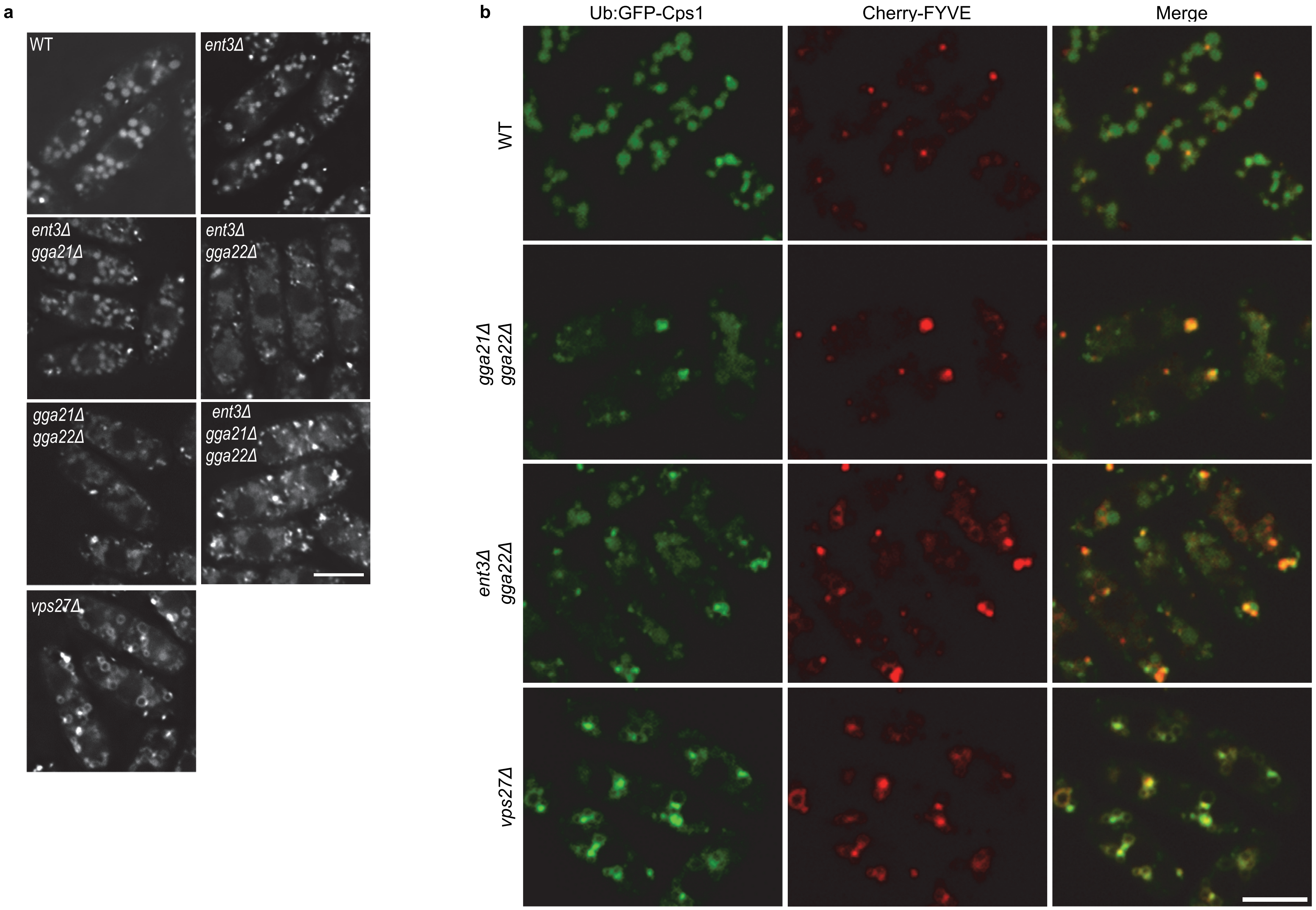

Figure S4. Distribution of Ub:GFP-Cps1. (a) Distribution of Ub:GFP-Cps1 in the indicated mutants. Images are medial planes captured with a DeltaVision system. **(b)** Colocalization between Ub:GFP-Cps1 and Cherry-FYVE in different strains. Images are medial planes captured with a confocal spinning-disk microscope. Bar, 10 μm.

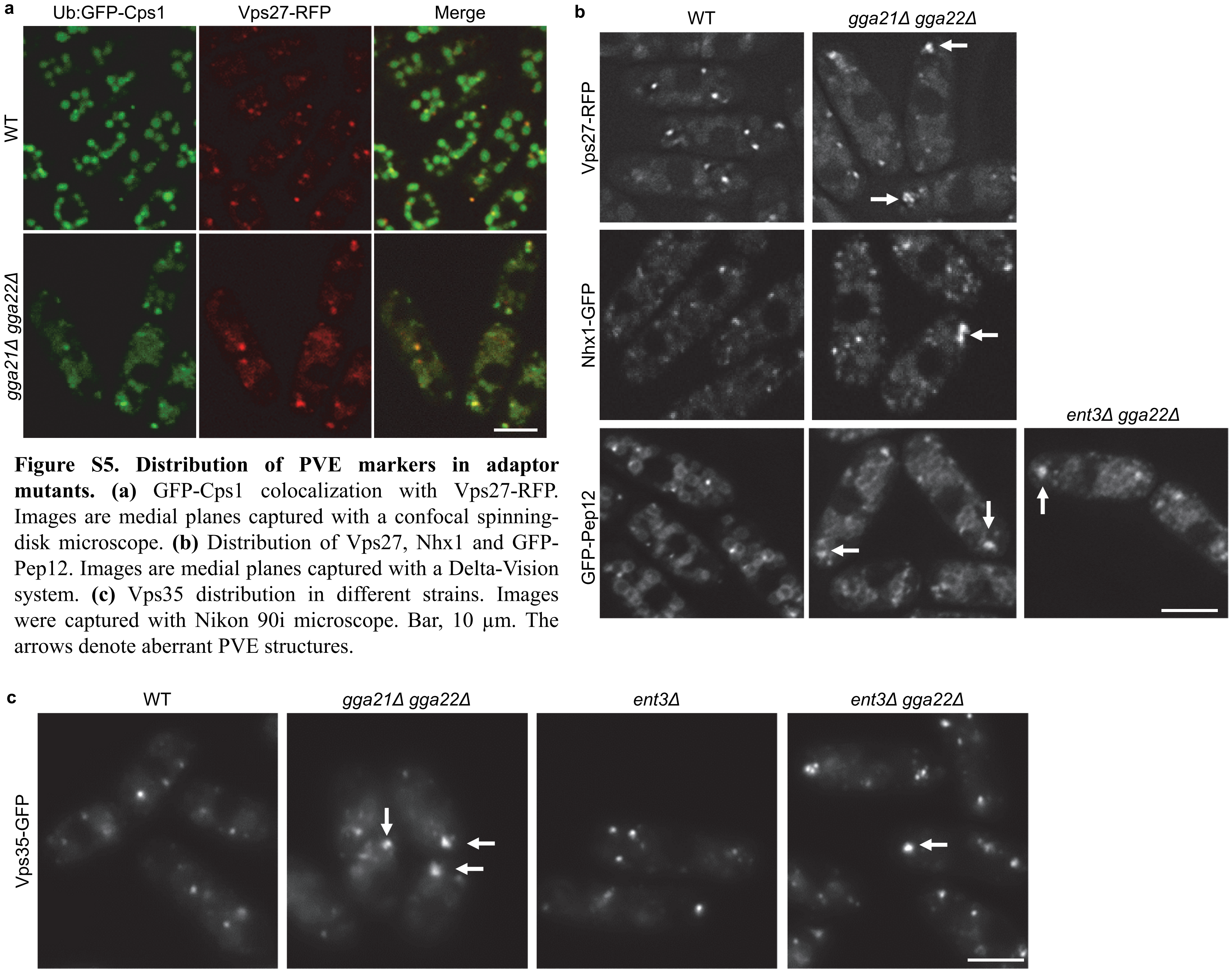

Figure S5. Distribution of PVE markers in adaptor mutants. **(a)** GFP-Cps1 colocalization with Vps27-RFP. Images are medial planes captured with a confocal spinning-disk microscope. **(b)** Distribution of Vps27, Nhx1 and GFP-Pep12. Images are medial planes captured with a Delta-Vision system. **(c)** Vps35 distribution in different strains. Images were captured with Nikon 90i microscope. Bar, 10 μm. The arrows denote aberrant PVE structures.

Supplementary Table S1

STRAIN	GENOTYPE	SOURCE
HVP30	*leu1-32 his3-Δ1 ura4-Δ18 ade6* h^{-}	Lab stock
HVP117	*leu1-32 his3-Δ1 ura4-Δ18 ade6* h^{+}	Lab stock
HVP1044	*cfr1::his3*$^{+}$ *cfr1-RFP:ura4*$^{+}$ *leu1-32 his3-Δ1 ura4-Δ18 ade6* h^{90}	Lab stock
HVP2092	*apm1::ura4 leu1-32* h^{-}	T Kuno
HVP2273	*GFP-syb1 leu1-32* h^{-}	Y Toyoshima
HVP3490	*apm1::ura4*$^{+}$ *his3-Δ1* h^{-}	Lab stock
HVP3664	*vps27::KAN leu1-32 ura4-Δ18 ade6* h^{+}	P Perez
HVP3701	*apm3::KAN leu1-32 ura4-Δ18 ade6* h^{+}	P Perez
HVP3727	*apm1::ura4*$^{+}$ *apm1-GFP:leu1*$^{+}$ *leu1-32 ura4-Δ18 his3-Δ1* h^{-}	Lab stock
HVP3945	*pku70::his3*$^{+}$ *leu1-32 his3-Δ1 ura4-Δ18 ade6* h^{+}	I Hagan / YGRC
HVP3953	*hph.171* h^{-}	I Hagan / YGRC
HVP3954	*gga21::KAN leu1-32 his3-Δ1 ura4-Δ18 ade6* h^{-}	Lab stock
HVP3956	*gga22::KAN leu1-32 his3-Δ1 ura4-Δ18 ade6* h^{-}	Lab stock
HVP3994	*gga21::KAN gga22::KAN his3-Δ1* h^{-}	Lab stock
HVP4022	*pik3::KAN leu1-32 ura4-Δ18 ade6* h^{+}	P Perez
HVP4068	*hph.171 leu1-32 his3-Δ1 ura4-Δ18* h^{+}	This work
HVP4160	*cfr1:.his3 cfr1-RFP:ura4*$^{+}$ *Pnda2:GFP-FYVE:Tnda2:NAT leu1-32 his3-Δ1 ura4-Δ18 ade6* $h^{?}$	This work
HVP4317	*gga22::KAN gga22-GFP:ura4*$^{+}$ *leu1-32 his3-Δ1 ura4-Δ18 ade6* h^{+}	Lab stock
HVP4373	*gga22::KAN GFP-syb1:KAN* $h^{?}$	Lab stock
HVP4374	*gga21::KAN gga22:.KAN GFP-syb1:KAN* $h^{?}$	Lab stock
HVP4243	*Pnda2:mCherry-FYVE:Tnda2:NAT leu1-32 his3-Δ1 ura4-Δ18* h^{+}	Lab stock
HVP4509	*ent3::KAN leu1-32 his3-Δ1 ura4-Δ18 ade6* h^{+}	P Perez
HVP4523	*gga22::KAN Pnda2:mCherry-FYVE:Tnda2):NAT* h^{+}	This work
HVP4525	*gga21::KAN gga22::KAN Pnda2:mCherry-FYVE:Tnda2:NAT* h^{+}	This work
HVP4637	*gga22::KAN gga22-RFP:ura4*$^{+}$ *Pnda2:GFP-FAPP1:Tnda2:NAT leu1-32 his3-Δ1 ura4-Δ18 ade6 h?*	This work
HVP4680	*clc1::KAN clc1-GFP:leu1*$^{+}$ *leu1-32 his3-Δ1 ura4-Δ18 ade6*$^{?}$ h^{+}	This work
HVP4708	*vps10-GFP:KAN leu1-32 his3-Δ1 ura4-Δ18 ade6* h^{-}	This work
HVP4709	*vps10-GFP:KAN leu1-32 his3-Δ1 ura4-Δ18 ade6* h^{+}	This work
HVP4714	*vps27-RFP:KAN leu1-32 his3-Δ1 ura4-Δ18 ade6* h^{+}	This work
HVP4715	*vps27-RFP:KAN leu1-32 his3-Δ1 ura4-Δ18 ade6* h^{-}	This work
HVP4730	*vps10-GFP:KAN gga21::KAN leu1-32 his3-Δ1 ura4-Δ18 ade6* h^{+}	This work
HVP4735	*vps10-GFP:KAN gga22::KAN leu1-32 his3-Δ1 ura4-Δ18 ade6* h^{+}	This work
HVP4737	*vps10-GFP:KAN gga21::KAN gga22::KAN leu1-32 his3-Δ1 ura4-Δ18 ade6* h^{+}	This work
HVP4746	*ent3::KAN gga22::KAN gga22-GFP:ura4*$^{+}$ *leu1-32 his3-Δ1 ura4-Δ18 ade6* h^{-}	This work
HVP4751	*vps10-GFP:KAN Pnda2:mCherry-FYVE:Tnda2:NAT leu1-32 his3-Δ1 ura4-Δ18* h^{+}	This work
HVP4752	*vps10-GFP:KAN Pnda2:mCherry-FYVE:Tnda2:NAT leu1-32 his3-Δ1 ura4-Δ18* h^{-}	This work
HVP4767	*vps10-GFP:KAN pik3::KAN leu1-32 his3? ura4-Δ18 ade6* $h^{?}$	This work
HVP4777	*ent3::KAN clc1::KAN clc1-GFP:leu1*$^{+}$ *leu1-32 his3-Δ1 ura4-Δ18 ade6*$^{?}$ $h^{?}$	This work
HVP4781	*ent3::KAN gga21::KAN leu1-32 his3? ura4-Δ18 ade6* h^{+}	This work
HVP4782	*ent3::KAN gga22::KAN leu1-32 his3? ura4-Δ18 ade6* h^{-}	This work
HVP4783	*ent3::KAN gga21::KAN gga22::KAN leu1-32 his3? ura4-Δ18 ade6* h^{+}	This work
HVP4788	*vps27::KAN ent3::KAN leu1-32 ura4-Δ18 ade6* h^{-}	This work
HVP4789	*apm1::ura4*$^{+}$ *apm1-GFP:leu1*$^{+}$ *leu1-32 ura4-Δ18 his3-Δ1 ade6*$^{?}$ $h^{?}$	This work
HVP4790	*vps10-GFP:KAN Pnda2:mCherry-FYVE:Tnda2:NAT gga21::KAN leu1-32 his3-Δ1 ura4-Δ18* $h^{?}$	This work
HVP4791	*vps10-GFP:KAN Pnda2:mCherry-FYVE:Tnda2:NAT gga22::KAN leu1-32 his3-Δ1 ura4-Δ18* $h^{?}$	This work

HVP4792	vps10-GFP:KAN Pnda2:mCherry-FYVE:Tnda2:NAT gga21::KAN gga22::KAN leu1-32 his3-Δ1 ura4-Δ18 $h^?$	This work
HVP4805	vps27::KAN gga21::KAN leu1-32 ura4-Δ18 ade6 h^-	This work
HVP4806	vps27::KAN gga21::KAN leu1-32 ura4-Δ18 ade6 h^+	This work
HVP4808	vps27::KAN gga21::KAN gga22::KAN leu1-32 ura4-Δ18 ade6 h^+	This work
HVP4814	vps10-GFP:KAN ent3::KAN leu1-32 his3? ura4-Δ18 ade6 h^-	This work
HVP4821	vps10-GFP:KAN vps35::NAT leu1-32 his3? ura4-Δ18 ade6 h^-	This work
HVP4824	ent3-GFP:KAN Pnda2:mCherry-FYVE:Tnda2:NAT leu1-32 his3-Δ1 ura4-Δ18 h^+	This work
HVP4826	ent3-GFP:KAN Pnda2:GFP-FAPP1:Tnda2:NAT leu1-32 his3-Δ1 ura4-Δ18 ade6 h^+	This work
HVP4834	vps10-GFP:KAN ent3::KAN gga21::KAN leu1-32 his3? ura4-Δ18 ade6 h^+	This work
HVP4836	vps10-GFP:KAN ent3::KAN gga22::KAN leu1-32 his3? ura4-Δ18 ade6 h^+	This work
HVP4839	vps10-GFP:KAN ent3::KAN gga21::KAN gga22::KAN leu1-32 his3? ura4-Δ18 ade6 h^-	This work
HVP4841	vps10-GFP:KAN gga22::KAN leu1-32 his3-Δ1 ura4-Δ18 $h^?$	This work
HVP4848	vps10-GFP:KAN vps27::KAN leu1-32 his3? ura4-Δ18 ade6 h^+	This work
HVP4850	vps10-GFP:KAN ent3::KAN vps27::KAN leu1-32 his3? ura4-Δ18 ade6 h^+	This work
HVP4866	vps10-GFP:KAN Pnda2:mCherry-FYVE:Tnda2:NAT ent3::KAN leu1-32 his3-Δ1 ura4-Δ18 h^-	This work
HVP4876	Pnda2:GFP-Pep12:Tnda2:NAT leu1-32 his3-Δ1 ura4-Δ18 h^+	This work
HVP4879	vph1-GFP:KAN leu1-32 his3-Δ1 ura4-Δ18 ade6 h^-	This work
HVP4880	vph1-GFP:KAN leu1-32 his3-Δ1 ura4-Δ18 ade6 h^+	This work
HVP4883	vph1-GFP:KAN ent3::KAN leu1-32 his3-Δ1 ura4-Δ18 ade6 h^+	This work
HVP4885	vph1-GFP:KAN gga22::KAN leu1-32 his3-Δ1 ura4-Δ18 ade6 h^+	This work
HVP4887	vph1-GFP:KAN ent3::KAN gga22::KAN leu1-32 his3-Δ1 ura4-Δ18 ade6 h^+	This work
HVP4892	vps10-GFP:KAN cfr1::his cfr1-RFP:ura4+ leu1-32 his3-Δ1 ura4-Δ18 ade6 h^+	This work
HVP4899	Pnda2:GFP-Pep12:Tnda2:NAT ent3::KAN leu1-32 his3-Δ1 ura4-Δ18 h^-	This work
HVP4917	vps10-GFP:KAN Pnda2:mCherry-FYVE:Tnda2:NAT ent3::KAN gga22::KAN leu1-32 his3-Δ1 ura4-Δ18 h^+	This work
HVP4926	vps35-GFP:NAT ent3::KAN pku70::$his3^+$ leu1-32 his3-Δ1 ura4-Δ18 ade6 h^-	This work
HVP4930	vps35-GFP:NAT ent3::KAN gga22::KAN pku70::$his3^+$ leu1-32 his3-Δ1 ura4-Δ18 ade6 h^-	This work
HVP4943	vps10-GFP:KAN cfr1::his cfr1-RFP:ura4+ ent3::KAN leu1-32 his3-Δ1 ura4-Δ18 ade6 h^+	This work
HVP4946	vps10-GFP:KAN cfr1::his cfr1-RFP:ura4+ gga22::KAN leu1-32 his3-Δ1 ura4-Δ18 ade6 h-	This work
HVP4951	vps27-RFP:KAN gga21::KAN gga22::KAN leu1-32 his3-Δ1 ura4-Δ18 ade6? h^-	This work
HVP4953	GFP-syb1:KAN Pnda2:mCherry-FYVE:Tnda2:NAT gga21::KAN gga22::KAN leu1-32 $his3^?$ $ura4^?$ $ade6^?$ $h^?$	This work
HVP5016	Pnda2:GFP-Pep12:Tnda2:NAT gga21::KAN gga22::KAN leu1-32 his3-Δ1 ura4-Δ18 $ade6^?$ h^-	This work
HVP5038	vps35-GFP:NAT leu1-32 his3-Δ1 ura4-Δ18 ade6 h^+	This work
HVP5039	vps35-GFP:NAT leu1-32 his3-Δ1 ura4-Δ18 ade6 h^-	This work
HVP5043	vps35-GFP:NAT gga21::KAN gga22::KAN leu1-32 his3-Δ1 ura4-Δ18 ade6 h^-	This work
HVP5058	vph1-GFP:KAN gga21::KAN leu1-32 his3-Δ1 ura4-Δ18 ade6 h^+	This work
HVP5059	vph1-GFP:KAN gga21::KAN gga22::KAN leu1-32 his3-Δ1 ura4-Δ18 ade6 h^-	This work
HVP5068	vph1-GFP:KAN vps27::KAN leu1-32 his3-Δ1 ura4-Δ18 ade6 h^+	This work
HVP5086	isp6::$ura4^+$ psp3::NAT h^-	M Cabrera / E Hidalgo
HVP5096	cpy1-mCherry:KAN leu1-32 his3-Δ1 h^-	L L Du
HVP5121	vps10-GFP:KAN psp3::NAT h^-	This work
HVP5122	vps10-GFP isp6::$ura4^+$ h^-	This work
HVP5123	vps10-GFP isp6::$ura4^+$ psp3::NAT h^-	This work
HVP5124	vps10-GFP:KAN vps35::NAT psp3::NAT	This work
HVP5125	vps10-GFP:KAN vps35::NAT isp6::$ura4^+$	This work
HVP5127	vps10-GFP vps35::NAT isp6::$ura4^+$ psp3::NAT	This work
HVP5128	Pnda2:ub:GFP-cps1:Tnmt1:NAT leu1-32 his3-Δ1 ura4-Δ18 ade6 h^-	This work

HVP5135	Pnda2:ub:GFP-cps1:Tnmt1:NAT gga21::KAN gga22::KAN leu1-32 his3-Δ1 ura4-Δ18 ade6 h^{-}	This work
HVP5152	Pnda2:mCherry-FYVE:Tnda2:NAT Pnda2:GFP-FAPP1:Tnda2:$leu1^{+}$ h^{+}	This work
HVP5153	vps10-GFP:KAN gga21::KAN gga22::KAN isp6:.$ura4^{+}$ h^{+}	This work
HVP5155	vps10-GFP:KAN gga21::KAN gga22::KAN psp3::NAT h^{+}	This work
HVP5157	vps10-GFP:KAN gga21::KAN gga22::KAN isp6:.ura4 psp3::NAT^{+} h^{-}	This work
HVP5163	Pnda2:ub:GFP-cps1:Tnmt1:NAT vps27-RFP:KAN leu1-32 his3-Δ1 ura4-Δ18 ade6 $h^{?}$	This work
HVP5165	Pnda2:ub:GFP-cps1:Tnmt1:NAT vps27-RFP:KAN gga21::KAN gga22::KAN leu1-32 his3-Δ1 ura4-Δ18 ade6 $h^{?}$	This work
HVP5168	Pnda2:ub:GFP-cps1:Tnmt1:NAT vps27::KAN leu1-32 his3-Δ1 ura4-Δ18 ade6 h^{-}	This work
HVP5185	Pnda2:mCherry-FYVE:Tnda2:NAT Pnda2:GFP-FAPP1:Tnda2:$leu1^{+}$ gga21::KAN gga22::KAN $h^{?}$	This work
HVP5200	nhx1-GFP:KAN leu1-32 his3-Δ1 ura4-Δ18 ade6 h^{-}	This work
HVP5204	nhx1-GFP:KAN gga21::KAN gga22::KAN leu1-32 his3-Δ1 ura4-Δ18 ade6 $h^{?}$	This work
HVP5205	Pnda2.ub:GFP-cps1:Tnmt1:NAT ent3::KAN leu1-32 his3-Δ1 ura4-Δ18 ade6 $h^{?}$	This work
HVP5206	vps10-GFP:KAN Pnda2:mCherry-FYVE:Tnda2:NAT apm1::ura4 leu1-32 his3-Δ1 ura4-Δ18 $h^{?}$	This work
HVP5212	vps10-GFP:KAN Pnda2:mCherry-FYVE:Tnda2:NAT ent3::KAN gga21::KAN gga22::KAN leu1-32 his3-Δ1 ura4-Δ18 $h^{?}$	This work
HVP5213	vps10-GFP:KAN cfr1::his cfr1-RFP:ura apm1::ura4 leu1-32 his3-Δ1 ura4-Δ18 ade6? $h^{?}$	This work
HVP5224	vps10-GFP:KAN cfr1::his cfr1-RFP:ura ent3::KAN gga21::KAN leu1-32 his3-Δ1 ura4-Δ18 ade6 $h^{?}$	This work
HVP5225	vps10-GFP:KAN cfr1::his cfr1-RFP:$ura4^{+}$ ent3::KAN gga22::KAN leu1-32 his3-Δ1 ura4-Δ18 ade6 $h^{?}$	This work
HVP5226	vps10-GFP:KAN cfr1::his cfr1-RFP:$ura4^{+}$ ent3::KAN gga21::KAN gga22::KAN leu1-32 his3-Δ1 ura4-Δ18 ade6 h^{-}	This work
HVP5227	Pnda2:ub:GFP-cps1:Tnmt1:NAT ent3::KAN gga21::KAN leu1-32 his3-Δ1 ura4-Δ18 ade6 h?	This work
HVP5228	Pnda2:ub:GFP-cps1:Tnmt1:NAT ent3::KAN gga22::KAN leu1-32 his3-Δ1 ura4-Δ18 ade6 h?	This work
HVP5229	Pnda2:ub:GFP-cps1:Tnmt1:NAT ent3::KAN gga21::KAN gga22::KAN leu1-32 his3-Δ1 ura4-Δ18 ade6 $h^{?}$	This work
HVP5230	isp6-GFP:KAN Pnda2:mCherry-FYVE:Tnda2:$leu1^{+}$ leu1-32 his3-Δ1 ura4-Δ18 ade6 $h^{?}$	This work
HVP5231	isp6-GFP:KAN Pnda2:mCherry-FYVE:Tnda2:$leu1^{+}$ gga21::KAN gga22::KAN leu1-32 his3-Δ1 ura4-Δ18 ade6 $h^{?}$	This work
HVP5253	vps10-GFP:KAN Pnda2:mCherry-FYVE:Tnda2:NAT apm3::KAN gga21::KAN gga22::KAN leu1-32 his3-Δ1 ura4-Δ18 $h^{?}$	This work
HVP5255	cfr1:.his3 cfr1-RFP:$ura4^{+}$ Pnda2:GFP-FYVE:Tnda2:NAT gga21::KAN gga22::KAN leu1-32 his3-Δ1 ura4-Δ18 ade6 h?	This work
HVP5267	Pnda2:ub:GFP-cps1:Tnmt1:NAT cfr1::his3 cfr1-RFP:$ura4^{+}$ leu1-32 his3-Δ1 ura4-Δ18 ade6 h^{-}	This work
HVP5268	Pnda2:ub:GFP-cps1:Tnmt1:NAT cfr1::his3 cfr1-RFP:$ura4^{+}$ ent3::KAN gga22::KAN leu1-32 his3-Δ1 ura4-Δ18 ade6 $h^{?}$	This work
HVP5269	Pnda2:ub:GFP-cps1:Tnmt1:NAT cfr1::his3 cfr1-RFP:$ura4^{+}$ gga21::KAN gga22::KAN leu1-32 his3-Δ1 ura4-Δ18 ade6 $h^{?}$	This work
HVP5270	Pnda2:ub:GFP-cps1:Tnmt1:NAT Pnda2:mCherry-FYVE:Tnda2:$leu1^{+}$ leu1-32 his3-Δ1 ura4-Δ18 $ade6^{?}$ h^{+}	This work
HVP5271	Pnda2:ub:GFP-cps1:Tnmt1:NAT Pnda2:mCherry-FYVE:Tnda2:$leu1^{+}$ vps27::KAN leu1-32 his3-Δ1 ura4-Δ18 $ade6^{?}$ $h^{?}$	This work
HVP5272	Pnda2:ub:GFP-cps1:Tnmt1:NAT Pnda2:mCherry-FYVE:Tnda2:$leu1^{+}$ gga21::KAN gga22::KAN leu1-32 his3-Δ1 ura4-Δ18 $ade6^{?}$ $h^{?}$	This work
HVP5273	Pnda2:ub:GFP-cps1:Tnmt1:NAT Pnda2:mCherry-FYVE:Tnda2:$leu1^{+}$ ent3::KAN gga22::KAN leu1-32 his3-Δ1 ura4-Δ18 $ade6^{?}$ $h^{?}$	This work
HVP5282	cpy1-mCherry:KAN gga21::KAN gga22::KAN leu1-32 his3-Δ1ura4? ade6? h^{-}	This work

HVP5294	*vps10-GFP:KAN ent3::KAN gga21::KAN gga22::KAN isp6::ura4$^+$ h^{90}*	This work
HVP5301	*vps10-GFP:KAN ent3::KAN gga22::KAN isp6::ura4$^+$ h$^-$*	This work
HVP5314	*vps10-GFP:KAN Pnda2:mCherry-FYVE:Tnda2:NAT ent3::KAN gga21::KAN leu1-32 his3-Δ1 ura4-Δ18 h$^-$*	This work
HVP5315	*Pnda2:GFP-Pep12:Tnda2:NAT ent3::KAN gga22::KAN leu1-32 his3-Δ1 ura4-Δ18 ade6? h$^+$*	This work
HVP5319	*GFP-syb1:KAN Pnda2:mCherry-FYVE:Tnda2:NAT ent3::KAN gga22::KAN leu1-32 his3? ura4? ade6? h$^?$*	This work
HVP5320	*GFP-syb1:KAN Pnda2:mCherry-FYVE:Tnda2:NAT ent3::KAN gga21::KAN gga22::KAN leu1-32 his3$^?$ ura4$^?$ ade6$^?$ h$^?$*	This work

Fig6a

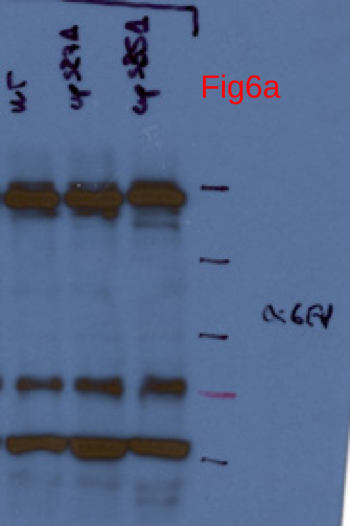

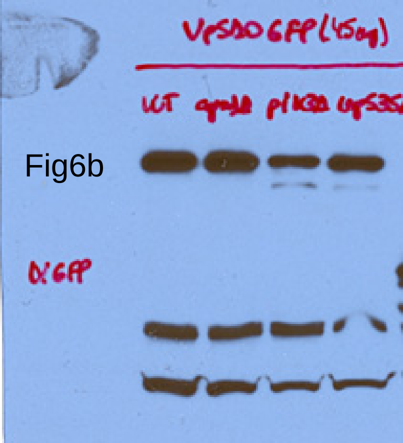
Fig6b

Fig6c

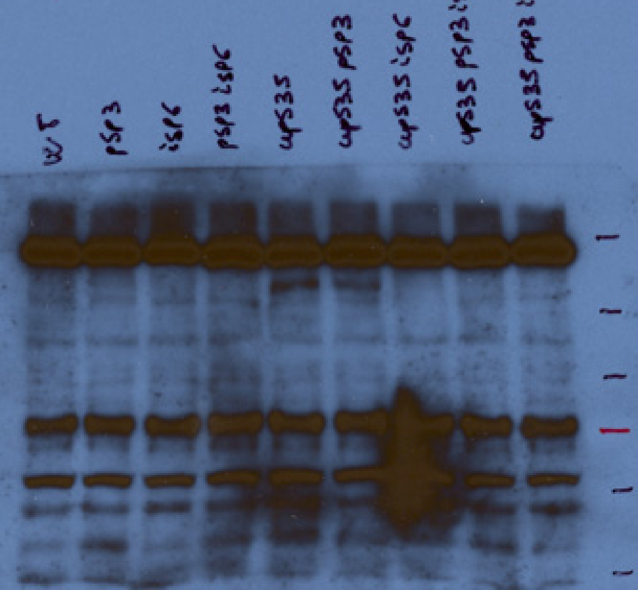
WT
psp3
isp6
psp3 isp6
vps35
vps35 psp3
vps35 isp6
vps35 psp3 isp6

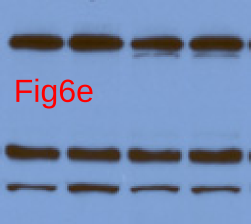
Fig6e

Fig6f UP

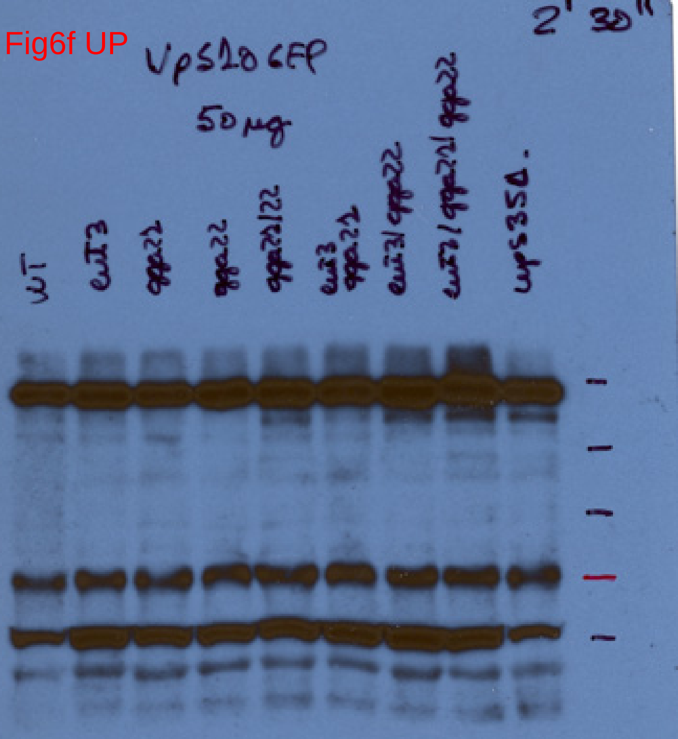

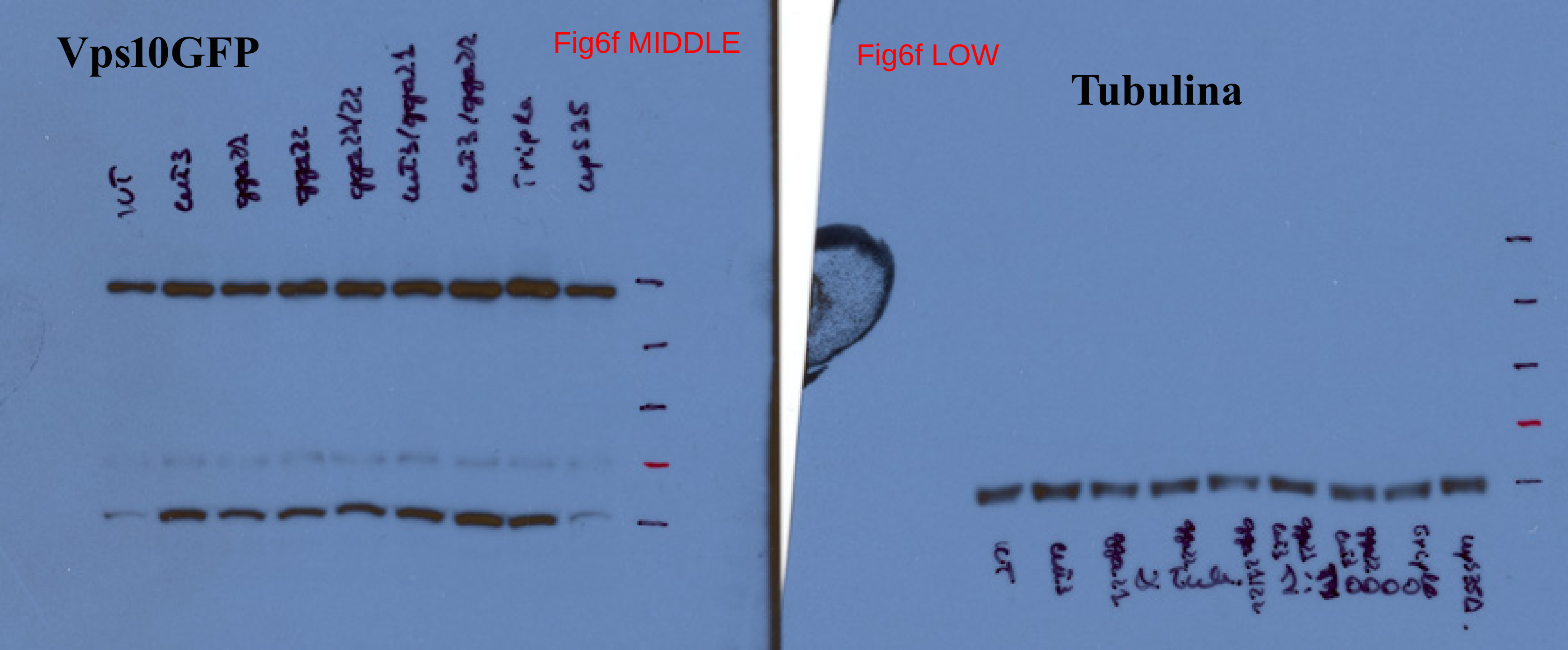
Vps10GFP
Fig6f MIDDLE
Fig6f LOW
Tubulina

Fig6g UP

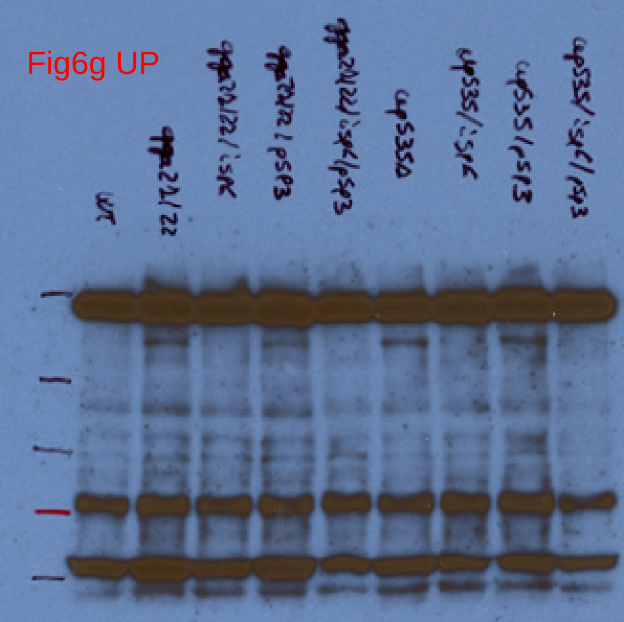

Fig6g LOW

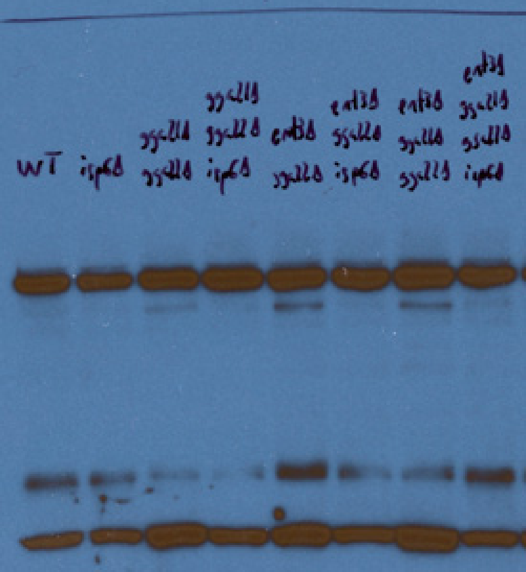

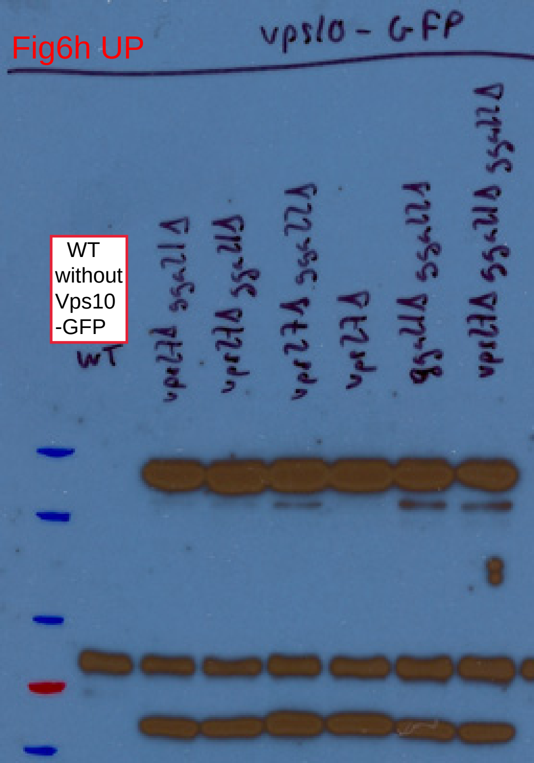
Fig6h UP
vps10 - GFP
WT without Vps10 -GFP
WT

Fig6h LOW

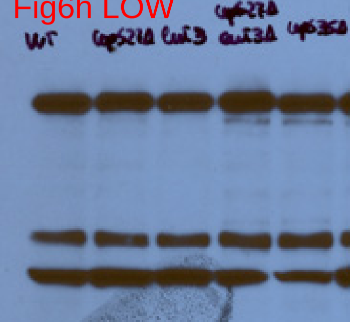

Supplement: Supplementary file 1 — Supplmentary information [file 41598_2019_47035_MOESM1_ESM.pdf]
